# The Comparative Efficacy of Multiple Acupuncture for Alzheimer's Disease: A Bayesian Network Meta-Analysis

**DOI:** 10.1155/2022/3288948

**Published:** 2022-05-17

**Authors:** Zihan Yin, Xiang Li, Linjia Wang, Mingsheng Sun, Ling Zhao, Fanrong Liang

**Affiliations:** ^1^School of Acu-Mox and Tuina, Chengdu University of Traditional Chinese Medicine, Chengdu, China; ^2^Acupuncture Clinical Research Center of Sichuan Province, Chengdu, China

## Abstract

**Background:**

Alzheimer's disease (AD) is a progressive neurodegenerative disease. Numerous cases have illustrated that the acupuncture method could improve AD patients' cognitive function and daily living ability. However, the optimal acupuncture treatments remain controversial. Therefore, we aimed to conduct a systematic review to compare the efficacy of multiple acupuncture therapies for AD and identify the optimal acupuncture intervention for delaying AD progression.

**Methods:**

To select potentially concerned randomized controlled trials (RCTs), we searched four English databases, four Chinese databases, and additional sources from 1 May 2021. Two independent reviewers conducted study screening, data extraction, and methodological quality assessment. The primary outcome was global cognitive function improvement. Pairwise and Bayesian network meta-analyses were performed using STATA v15.0 and ADDIS v1.16.8. The Grading of Recommendations Assessment, Development, and Evaluation (GRADE) tool was used to assess the quality of evidence.

**Results:**

This study included 34 RCTs with 2,071 participants. Regarding global cognitive function improvement, the pairwise meta-analysis confirmed that electronic acupuncture (EA) plus conventional medicine (CM) and manual acupuncture (MA) plus CM were statistically significantly different from CM, and EA plus CM was ranked as the best combination in the network meta-analysis. In terms of response rate, MA outperformed CM statistically significantly; warm acupuncture (WA) was ranked as the best in the network meta-analysis. Regarding activity of daily living improvement, EA plus CM, MA plus CM, and fire acupuncture plus CM, MA, and scalp acupuncture were statistically significantly different from CM, and EA plus CM was ranked as the best combination in the network meta-analysis. However, the evidences were ranked as low to critically low.

**Conclusions:**

Acupuncture, as a monotherapy or an adjuvant therapy, may have a beneficial effect on efficacy for AD. EA plus CM may be the optimal acupuncture therapy for AD and should be administered to AD patients. It may aid and support patient, operative, and societal decision-making. Due to the dearth of high-quality evidence, additional high-quality studies should be conducted to ensure these findings in the future. This study is registered with PROSPERO (CRD42021252305).

## 1. Introduction

Alzheimer's disease (AD), the most prevalent disorder of dementia, is characterized by tau and amyloid *β* (A*β*) accumulation [1, 2]. The disorder is manifested by a progressive decline of cognitive function [3]. Moreover, AD may negatively affect the activity of daily living (ADL) and psychological and behavioral conditions [4]. It is estimated that approximately 44 million people suffer from AD [5]. As the aging population proliferates worldwide, AD prevalence increases [6, 7], particularly in China, which has experienced exponential growth [8, 9]. In addition, it leads to a huge financial burden associated with AD control [9, 10].

Besides, because AD's mechanism remains unclear, there is no specific remedy for the whole AD process [11]. Current interventions for AD included several conventional medicines (CM), which have been approved by the US Food and Drug Administration (FDA), such as acetylcholinesterase inhibitors (AChEIs), N-methyl-D-aspartate (NMDA) receptor, and monoclonal antibody [12]. However, some evidences illustrated that their effect was negligible [13, 14]. Consequently, it is indispensable to seek new effective treatments for AD.

Notably, acupuncture has a long history of managing dementia [15, 16]. Several systematic reviews and meta-analyses demonstrated that acupuncture was not inferior to pharmacotherapy in treating AD [17–20]. Furthermore, numerous articles have indicated that acupuncture may improve cholinergic neurotransmission, decrease A*β* protein concentration, and stimulate exciter motor-related brain regions associated with cognitive function [21–23]. However, several acupuncture treatments have been applied for AD, including manual acupuncture (MA), electronic acupuncture (EA), fire acupuncture (FA), warm acupuncture (WA), scalp acupuncture (SA), and so on. Because the most effective acupuncture technique is unknown, seeking the optimal acupuncture intervention for AD is critical.

This study aimed to conduct a Bayesian network meta-analysis (NMA) [24] to drastically compare and rank various acupuncture therapies for AD in improving global cognitive function and activity of daily living. Meanwhile, our findings provided a new reference for clinical decision-making regarding acupuncture for AD.

## 2. Methods

The NMA is registered on the PROSPERO platform (number: CRD42021252305) and reported following the preferred reporting items for systematic reviews and meta-analyses (PRISMA-NMA) checklist [25].

### 2.1. Eligibility and Exclusion Criteria

#### 2.1.1. Types of Studies

This study included all randomized controlled parallel trials published in English/Chinese, regardless of region or publication restriction. By the way, the randomized cross-over trials' first period would be covered. On the other hand, randomized controlled cluster trials, case reports, experts' experience, and so on were excluded.

#### 2.1.2. Types of Participants

This study included all participants with definite AD diagnostic criteria, regardless of their gender, country, ethnic origin, or severity. Participants with dementia who did not have a precise AD diagnosis were excluded.

#### 2.1.3. Types of Intervention

MA, EA, WA, FA, and SA are high-frequency acupuncture therapies. These acupuncture treatments were regarded as either monotherapies or integrative therapies. Moreover, integrative treatments combining acupuncture and CM would be covered. The Chinese herb, exercise, music therapies, and so on would be excluded.

#### 2.1.4. Types of Control Groups

Numerous acupuncture treatments and CM (AChEIs, such as donepezil, huperzine, and rivastigmine) were used to form the basis.

#### 2.1.5. Types of Outcome Measures

We included studies that addressed one or more of the below-highlighted outcomes. Our primary outcome measure for NMA is global cognitive function improvement, as determined by Mini-Mental State Examination (MMSE) and Alzheimer's Disease Assessment Scale-Cognitive (ADAS-cog). Secondary outcomes included response rate and improvement in activity of daily living (ADL) as measured by ADL scales. Meanwhile, adverse events (AEs) would be included to measure intervention safety. Other outcomes of AD would be eliminated.

### 2.2. Search Strategy

From inception to 1 May 2021, the following databases were searched for acupuncture for AD: Cochrane Central Register of Controlled Trials (CENTRAL), PubMed, Embase, Web of Science (WOS), China National Knowledge Infrastructure (CNKI), Chinese Biomedical Literature Database (CBM), China Science and Technology Journal Database (VIP), and WanFang Database (WF). Additionally, additional sources were used as supplements, such as World Health Organization International Clinical Trials Registry Platform (WHO ICTRP), Clinical Trials.gov, Chinese Clinical Trial Register (ChiCTR), Grey Literature Database, and reported meta-analyses about acupuncture for AD. The search model consisted of both subject and random terms. The following terms were used in the search: (1) disease: Alzheimer disease, Alzheimer's disease, AD, and so on; (2) acupuncture intervention: acupuncture, acupuncture therapy, manual acupuncture, electronic acupuncture, warm acupuncture, fire acupuncture, moxibustion, and so on; and (3) study types: randomized controlled trials or RCTs. Various search strategies are presented in Table S1.

### 2.3. Study Selection and Data Extraction

Two reviewers (ZY and LW) were trained on a professional course on NMA. ZY and LW independently screened titles, abstracts, and keywords to identify duplicate trials and clearly ineligible studies and then excluded them. Following that, the full text of the studies was examined to ensure that they met inclusion criteria. If no ideal solution exists, the referee (LZ or FL) would make the final decision.

Two independent investigators (ZY and LW) extracted data using a six-part standardization extraction form: (1) identification information (publication year and first author), (2) general information (language, sample size, allocation ratio, diagnostic criteria, age, gender, course of disease, and severity of disease), (3) details of the acupuncture group (according to Revised Standards for Reporting Interventions in Clinical Trials of Acupuncture (STRICTA) [26], (4) details of the control group, (5) outcomes, and (6) main results. The selection procedure is depicted using a PRISMA flow graph.

### 2.4. Study Quality Assessment

The risk of bias (ROB) of each study was independently evaluated by two assessors using Cochrane Handbook [27]. This Cochrane ROB Tool comprised seven parts (random sequence generation, allocation concealment, blinding of participants and personnel, blinding of outcome assessors, incomplete outcome data, selective reporting, and other bias) and ranked the methodological quality as unclear, low, or high. A third party (LZ or FL) was consulted and aided in the final decision-making process. The ROB plot was generated using ReviewManager (RevMan) version 5.4 software (Cochrane, London, UK).

### 2.5. Statistical Analysis

#### 2.5.1. Pairwise Meta-Analysis

The pairwise meta-analysis was conducted using STATA software version 15.0 (Stata Corp LP, College Station, Texas, USA). The pre-post differences or end-point scores were calculated as outcomes. For the meta-analysis, three-arm trials were divided into two two-arm trials. The Mantel–Haenszel method used a fixed-effects model, whereas Der Simonian–Laired method utilized a random-effects model. The statistical heterogeneity was identified and measured by *I*^*2*^ statistics and *p*-value. The risk ratios (RR) were used for dichotomous data with a 95% confidence interval (CI). For continuous with 95% CI, weighted mean differences (WMD)/mean differences (MD) were applied. Based on guidelines from Cochrane Handbook 5.4, we deemed no statistical heterogeneity when *I*^2^<50% and *p* > 0.05.

#### 2.5.2. Network Meta-Analysis

STATA V15.0 was used to generate network plots of various treatment comparisons for each outcome. Aggregate Data Drug Information System (ADDIS V.1.16.8, Drugis, Groningen, NL) was applied to generate Bayesian NMA using Markov chain Monte Carlo (MCMC) algorithm [28]. Meanwhile, ADDIS V.1.16.8 was employed to generate indirect and direct comparisons. Using node-splitting analysis, ADDIS models were separated into consistency and inconsistency models. All nodes had *p*-values ≥0.05, indicating no statistically significant difference between indirect and direct comparisons, and we may employ the consistency model. The consistency model was used to determine the probability ranking of the best treatment for each outcome. The model's convergence is indicated by the potential scale reduced factor (PSRF). If the PSRF value was less than 1.2, it would be considered acceptable. For each acupuncture method, the ranking probabilities were generated in each outcome.

### 2.6. Publication Bias

As the analysis included over 10 RCTs, we used a comparison-adjusted funnel graph to assess reporting bias. If the included studies were symmetrically distributed on either side of the midline, there is a low risk of reporting bias.

### 2.7. Quality of Evidence

Using Grades of Recommendations, Assessment, Development, and Evaluation (GRADE) [29, 30], the overall quality of evidence was assessed and ranked as high, moderate, low, and critically low.

## 3. Results

### 3.1. Study Selection

**Figure 1:**
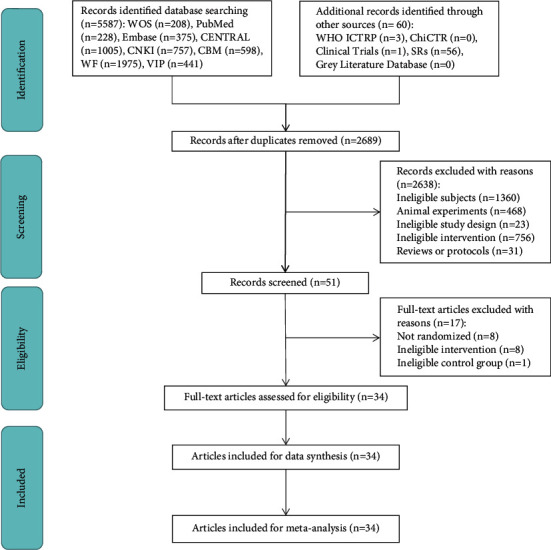
The PRISMA flow chart of the selection process.

Following a comprehensive search, 5,647 potential trials were identified. After removing duplicate trials, 2,689 studies remained. After initial screening, 51 articles remained. Finally, after reading the full text of the articles, 17 articles were excluded (8 non-RCT, 8 ineligible intervention groups, and 1 ineligible control group), and 34 RCTs remained [31–64]. The selection process is displayed in Figure 1, and the excluded full-text studies with reasons are listed in Table S2.

### 3.2. Study Characteristics

All included studies were implemented in China. The 34 studies were published between 2002 and 2021, with 2,071 patients; 30 trials were reported in Chinese, and 4 [44–46, 51] were published in English. Most studies had sample sizes of less than 100, and only 2 RCTs [31, 59] were equal to or greater than 100. The treatments of included studies consisted of MA, EA, WA, FA, SA, and CM, as well as integrations between these acupuncture therapies or with CM. Donepezil was the most frequently used medication in control groups. The allocation ratio was 1:1 or nearly 1:1. The frequently used diagnostic criteria were the Diagnostic and Statistical Manual of Mental Disorders (DSM) criteria. However, in the past 5 years, the National Institute on Aging and Alzheimer's Association (NIA-AA) criteria were commonly used. Besides, the mean age of AD participants was 60 to 80 years, and the number of males was less than that of females. The course of the disease mainly ranged from 3 to 5 years. Only 7 studies [41, 44,46, 56–58, 62] reported on the severity of AD. The treatment duration in the included studies ranged from 28 to 84 days. MMSE score improvement was the most mentioned outcome.  Table 1 summarizes the major characteristics of all included RCTs.

### 3.3. Acupuncture Details

As determined by the STRICTA tool, the details of acupuncture methods were extracted and displayed in Table 2. All included trials referred to acupuncture rationale. In needling details, the number of needle insertions per subject per session of the 34 trials mainly ranged from 8 to 10; the frequently used acupoints for AD were Baihui (DU 20), Zu San Li (ST 36), and Sishencong (EX-HN1); the acupuncture insertion depth varied widely due to different acupoints; a total of 13 studies [36, 38, 40, 41, 49, 51–55, 60, 62, 63] were lacking in response sought; the commonly used needle stimulation was manual acupuncture; the generally frequently used acupuncture brand was Hwato, as well as the diameter and length of acupuncture were 0.35 and 40 mm, respectively. The number of treatment sessions was various in the treatment regimen, and the frequency of treatment sessions was 5 to 6 times per week. In other components, only 2 trials [33, 56] covered the details of other interventions. In practitioners, only 3 studies [32, 34, 44] covered acupuncturists' details. In comparator interventions, more than half of included articles reported rationale for control/comparator, while two studies [37, 38] did not clearly illustrate the control/comparator.

### 3.4. Quality Assessment

**Figure 2:**
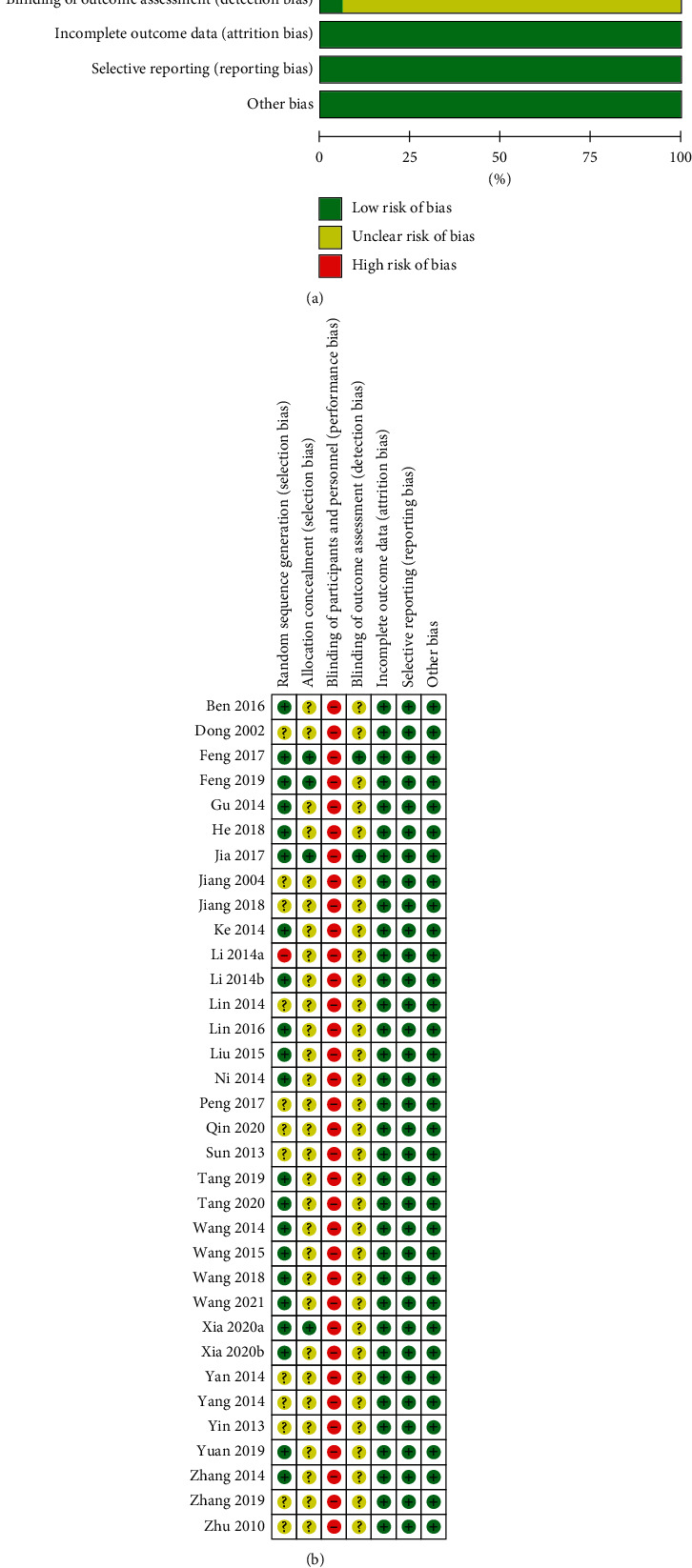
(a) Risk of bias graph and (b) risk of bias summary.

ROB of included trials was evaluated using Cochrane ROB Assessment Tool v.5.4. Although all 34 RCTs were reported using a random method, 12 trials were unclearly reported in random sequence generation, and 1 study [50] was grouped by date. Only 4 studies [34, 39, 43, 44] produced low risk in allocation concealment. Due to acupuncture's exceptionality, a high risk of performance bias existed. In 2 trials, the method of blinding outcome assessors was successfully implemented [43, 44]. All included trials exhibited a low ROB in other parts.  Figure 2 illustrates the ROB results.

### 3.5. Pairwise Meta-Analysis Results

#### 3.5.1. Primary Outcome

(1) Global cognitive function*: improvement of MMSE.* We performed 10 classic pairwise meta-analyses using a random-effects model to compare the effectiveness of various acupuncture therapies with CM.  Table 3 details the results. EA + CM (2 RCTs, WMD, 5.56; 95% CI: 2.10–9.03), MA + CM (5 RCTs, WMD, 2.43; 95% CI: 0.78–4.07), and FA + CM (1 RCT, WMD, 4.14; 95% CI: 3.10–5.18) were highly statistically efficient than CM in improving MMSE. WA and MA revealed statistically significant differences (2 RCTs, WMD, 0.51; 95% CI: 0.02–1.00). No significant differences were observed between 5 acupuncture treatments (SA + CM, WA + CM, EA, MA, and SA) and CM, SA + CM, and SA.

(2) Global cognitive function*: reduction in ADAS-cog.* Herein, six classic pairwise meta-analyses were conducted using a random-effects model to compare the effectiveness of various acupuncture therapies with CM. The details are listed in Table 4. EA + CM (one RCT, WMD, 4.32; 95% CI: 1.55–7.09), MA + CM (three RCTs, WMD, 2.46; 95% CI: 1.12–3.80), and MA (three RCTs, WMD, 3.11; 95% CI: 1.74–4.47) were highly statistically efficient in reducing ADAS-cog than CM. SA + CM and SA demonstrated statistically significant differences (one RCT, WMD, 4.50; 95% CI: 2.18–6.82). No significant differences were identified between the two acupuncture treatments (SA + CM and SA) and CM.

#### 3.5.2. Secondary Outcome

(1) *Response Rate.* We performed 10 classic pairwise meta-analyses using a random-effects model to compare the effectiveness of various acupuncture therapies with CM.  Table 5 displays the details of the results. MA (6 RCTs, RR, 1.25; 95% CI: 1.02–1.54) was highly statistically efficient in response rate compared to CM. No significant differences were observed between the 7 acupuncture treatments (EA + CM, MA + CM, SA + CM, WA + CM, FA + CM, EA, and SA) and CM, WA and MA, SA + CM, and SA.

(2) *Improvement in ADL.* Herein, we generated 9 classic pairwise meta-analyses using a random-effects model to compare the effectiveness of various acupuncture therapies with CM.  Table 6 contains all details. EA + CM (1 RCT, WMD, 8.01; 95% CI: 3.23–12.79), MA + CM (5 RCTs, WMD, 3.90; 95% CI: 2.29–5.52), FA + CM (1 RCT, WMD, 1.63; 95% CI: 0.11–3.15), MA (7 RCTs, WMD, 1.92; 95% CI: 1.31–2.52), and SA (3 RCTs, WMD, 3.17; 95% CI: 1.49–4.85) were highly statistically efficient in improving ADL compared to CM. WA (2 RCTs, WMD, 1.82; 95% CI: 1.15–2.49) was highly statistically efficient in improving ADL than MA. SA + CM and SA manifested statistically significant differences (1 RCT, WMD, 4.90; 95% CI: 2.06–7.74). No significant differences were observed between the two acupuncture treatments (SA + CM, and EA) and CM.

### 3.6. Network Meta-Analysis Results

#### 3.6.1. Network Plot for Different Interventions

**Figure 3:**
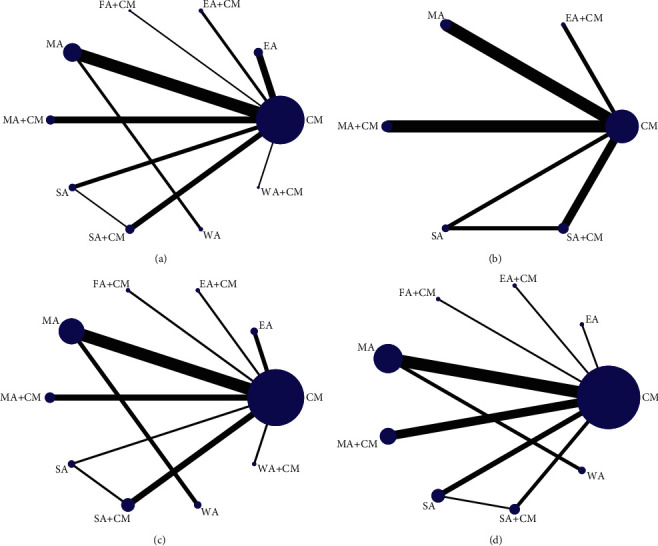
(a) The network graph of different interventions of improvement of MMSE, (b) the network graph of different interventions of reduction of ADAS-cog, (c) the network graph of different interventions of response rate, and (d) the network graph of different interventions of improvement of ADL.

We conducted 4 network plots using STATA 15.0. The line thickness is proportional to the 2 therapies, and the point size is positively correlated with the treatment sample size in Figure 3. MMSE improvement was reported in 31 studies involving 10 therapies and 1,874 subjects (Figure 3(a)), whereas ADAS-cog reduction was reported in 9 RCTs involving 708 patients and 6 interventions (Figure 3(b)). The response rate was revealed in 19 studies with 1,206 participants and 10 methods (Figure 3(c)). ADL improvement was reported in 21 RCTs with 1,366 patients and 9 interventions (Figure 3(d)).

#### 3.6.2. Evaluating Statistical Inconsistency

The node-splitting method was used to test the local inconsistency in MMSE improvement (Table S3(a)) and ADL (Table S3(b)). We found that *p* ≥ 0.05, demonstrating no significant difference between direct and indirect evidences. Due to no indirect evidence of a reduction in ADAS-cog and response rate, we performed a model of consistency.

#### 3.6.3. Evaluating Convergence of Consistency Model

According to PSRF results (all PSRF-value ≤1.2) in MMSE improvement (Table S4(a)), ADAS-cog reduction (Table S4(b)), response rate (Table S4(c)), and ADL improvement (Table S4(d)), the consistency model's convergence was acceptable.

#### 3.6.4. Primary Outcome

**Figure 4:**
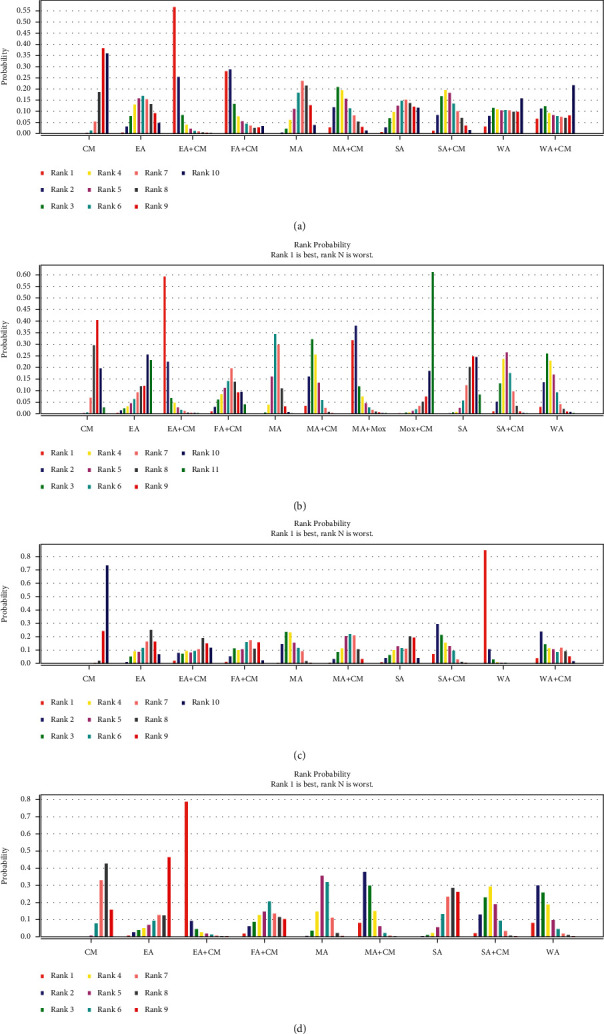
(a) The figure of the ranking probability of improvement of MMSE, (b) the figure of the ranking probability of reduction of ADAS-cog, (c) the figure of the ranking probability of response rate, and (d) the figure of the ranking probability of improvement of ADL.

(1) *Improvement of MMSE.*Table 7 illustrates the effect of NMA on MMSE improvement. In terms of efficacy, EA + CM outperformed MA (MD: 4.43; 95% CI: 0.04–8.70) and CM (MD: 5.49; 95% CI: 1.51–9.41). Based on Figure 4(a), EA + CM was proved the optimal acupuncture intervention in improving the MMSE score of 10 therapies in this NMA.

(2) *Reduction in ADAS-cog.* The NMA in ADAS-cog reduction is displayed in Table 8. In terms of efficacy, SA + CM (MD: 4.70; 95% CI: 0.76–8.20) and MA (MD: 3.21; 95% CI: 0.62–6.28) outperformed CM. Based on Figure 4(b), EA + CM was proved to be the optimal acupuncture intervention in reducing the ADAS-cog score of six therapies in this NMA.

#### 3.6.5. Secondary Outcome

(1) *Response rate.* The NMA response rate is displayed in Table 9. In terms of efficacy, WA (RR: 17.32; 95% CI: 5.47–42.46), WA + CM (RR: 4.23; 95% CI: 1.04–22.97), MA + CM (RR: 3.13; 95% CI: 1.62–6.96), SA + CM (RR: 5.53; 95% CI: 2.40 to 16.59), and MA (RR: 4.44; 95% CI: 2.52–7.63) outperformed CM. WA was significantly more effective than MA + CM (RR: 5.44; 95% CI: 1.30–16.01), FA + CM (RR: 5.70; 95% CI: 1.05–23.05), MA (RR: 3.86; 95% CI: 1.45–9.02), EA (RR: 7.21; 95% CI: 1.77–33.77), and SA (RR: 6.53; 95% CI: 1.24–28.38). Based on Figure 4(c), WA was proved as the optimal acupuncture intervention in response rate of 10 methods in this NMA.

(2) *Improvement in ADL.* The NMA for ADL improvement is displayed in Table 10. In terms of efficacy, EA + CM (MD: 7.94; 95% CI: 0.86–15.02), SA + CM (MD: 3.14; 95% CI: 0.54–5.90), MA + CM (MD: 4.26; 95% CI: 1.81–6.83), MA (MD: 1.94; 95% CI: 0.07–3.76), and WA (MD: 3.97; 95% CI: 0.41–7.63) outperformed CM. EA + CM (MD: 7.94; 95% CI: 0.86–15.02) and MA + CM (MD: 4.30; 95% CI: 0.39–8.09) outperformed SA. Based on Figure 4(d), EA + CM was proved as the optimal acupuncture intervention in response rate of 11 methods in this NMA.

### 3.7. Safety

Notably, six studies [31, 35, 36, 39, 43, 44] detailed the AEs of included treatments (Table 11). The medical methods included MA + CM, FA + CM, EA + CM, EA, MA, and CM. Acupuncture induced pain and a local hematoma. Meanwhile, CM mainly caused diarrhea, nausea, and emesis. None of the severe AEs was reported.

### 3.8. Heterogeneity

Acupuncture techniques, methods, acupuncture points, treatment duration, and other factors were different, resulting in high clinical heterogeneity. Therefore, we performed a sensitivity analysis using STATA 15.0 to assess the stability of the results, and we found that most combined effects were comparatively minor, and the results were reliable.

### 3.9. Publication Bias

**Figure 5:**
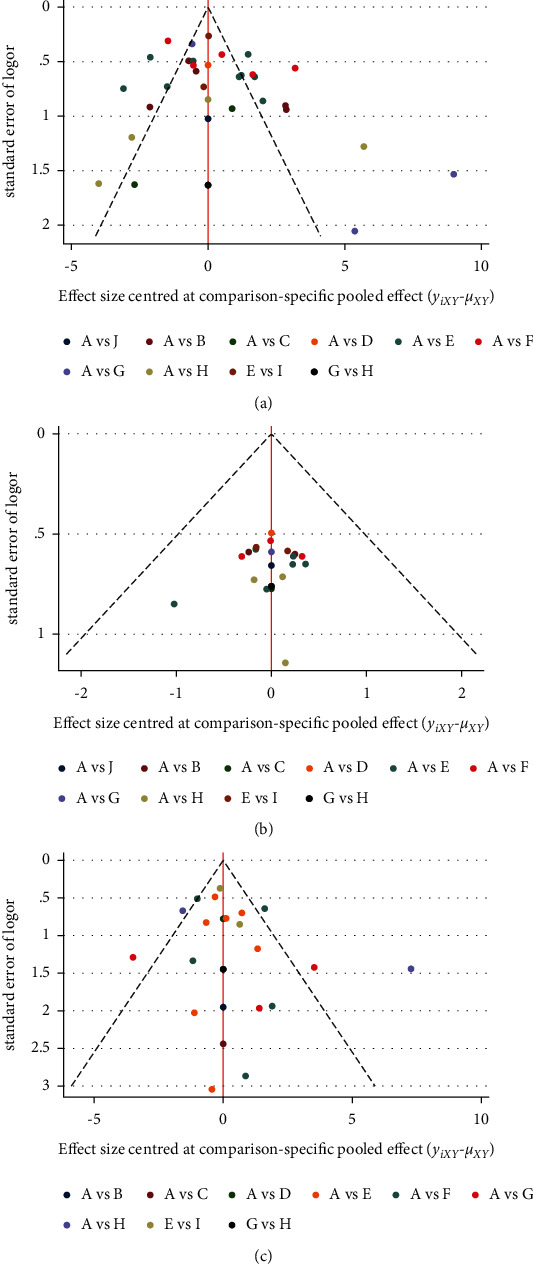
(a) Funnel plot for the network meta-analysis of improvement of MMSE, (b) funnel plot for the network meta-analysis of response rate, and (c) funnel plot for the network meta-analysis of improvement of ADL.

The reporting bias was assessed by comparing the symmetry of the comparison-adjusted funnel graph. Based on funnel plots regarding MMSE improvement (Figure 5(a)), response rate (Figure 5(b)), and ADL improvement (Figure 5(c)), most included studies were symmetrically distributed on either side of the midline, demonstrating that the likelihood of small sample effects was reduced. In ADAS-cog reduction, since the number of included RCTs did not exceed 10, funnel plots were not used to evaluate publication bias.

### 3.10. Quality of Evidence

According to the GRADE tool, the quality of the four outcomes (improvement in MMSE, ADAS-cog, ADL, and response rate) was low to critically low. Due to ROB, inconsistency, and imprecision, most evidence was rated critically low. Tables S5–S8 contain details about the evidence's quality.

## 4. Discussion

AD presents a remarkable public health problem, but FDA has approved only a few medical therapies, which could not affect the disease process [65, 66]. Numerous studies [17–20] proved that the acupuncture method effectively improved AD cognitive function and daily life ability. Some reviews [67, 68] demonstrated that acupuncture could induce neural plasticity, cell communication, regeneration, and gene expression in AD. Meanwhile, these studies have provided a mechanistic basis for acupuncture's efficacy in AD treatment. While various acupuncture therapies are applied nowadays, these have not been normalized or standardized. Doctors are compelled to use diversified acupuncture interventions, which imposes significant manpower and high economic burdens. Therefore, this NMA aims to identify optimal acupuncture therapy for AD using the most comprehensive information.

This meta-analysis aims to determine the efficacy of multiple acupuncture methods for AD treatment. The primary outcomes were shown as follows: (1) for MMSE improvement, EA + CM, MA + CM, and FA + CM efficacies were statistically different compared with CM efficacy; EA + CM was regarded as the optimal acupuncture method for MMSE improvement. (2) Meanwhile, for ADAS-cog reduction, when EA or MA was combined with CM, a high reduction in ADAS-cog was observed compared with CM alone; EA plus CM was deemed the most efficient acupuncture treatment. The results of secondary outcomes were summarized as follows: (1) regarding response rate, we discovered remarkable differences between MA and CM; WA was considered the optimal acupuncture therapy. (2) In improving ADL, EA + CM, MA + CM, FA + CM, MA, and SA efficacies were statistically different compared with CM efficacy; EA + CM was regarded as the optimal acupuncture method for ADL improvement. Moreover, six trials (16.22%) reported the safety of acupuncture-related AEs (pain, local hematoma, etc.). No severe AE existed. However, the GRADE tool determined that the overall quality of evidences from included studies was critically low.

This study has several strengths. (1) This is the first network meta-analysis comparing different acupuncture methods. Moreover, the optimal acupuncture method for AD treatment was identified, and (2) this NMA was strictly accomplished according to international standards. For instance, the report followed PRISMA-NMA guidelines, and acupuncture details met STRICTA criteria. (3) While numerous previous studies have examined efficacy outcomes, they lack objective and uniform standards. In this systematic review, the internationally acknowledged and commonly used measurement tools for cognitive function, MMSE and ADAS-cog, were regarded as the primary outcome indicators. ADL scale was applied as a secondary outcome indicator to assess improvement in activity function. In addition, the clinical efficacy of AD was further illustrated using response rate and adverse events. (4) To ensure the robustness of the results, we conducted a sensitivity analysis. (5) The quality of evidence is critical for clinical decision-making, which can be assessed using GRADE.

Meanwhile, this systematic review has some limitations. First, all included trials were conducted in China, which may introduce regional bias. In addition, the sample size of included trials was small, which may cause insufficient statistical efficiency. Third, because numerous acupuncture articles did not adhere to the STRICTA statement, they may leave many important details. Besides, the methodological and evidence quality of included studies was low, which could impair the findings' reliability and efficiency. Fifth, as is known to all, AD can be divided into mild, moderate, and severe periods. Since most of the RCTs did not provide sufficient AD stage information, we did not accomplish the analyses to explore the various stage differences in the curative effect of acupuncture interventions. Next, although this study had limited intervention methods in detail, non-standard factors contributed to clinical heterogeneity in acupuncture and CM. Additionally, while acupuncture is known for its long-term effects, this study focused exclusively; this study only concentrated on short-term efficacy and lacked long-term efficacy.

Numerous recommendations for future research exist. (1) In terms of methodological quality of the included trial, the influencing factors leading to poor quality are randomization, allocation concealment, and blinding; ROB, inconsistency, and imprecision resulted in degradation in evidence quality. Therefore, future studies should strictly adhere to the latest edition of the Cochrane Handbook for Systematic Reviews and the GRADE tool. (2) Besides, numerous investigations failed to report acupuncture details in a standardized approach in acupuncture trials. Thus, Consolidated Standards of Reporting Trials (CONSORT) [69, 70] and STRICTA were proposed to govern the reporting. (3) Clinical heterogeneity was observed due to various factors, including acupoint selection, treatment duration, CM dose, and so on. Therefore, the acupuncture industry should not only seek the optimal acupuncture methods but also address the optimal acupoints, treatment time, and frequency for AD treatment. (4) Additionally, future acupuncture studies for AD should focus on both short- and long-term efficacy. Besides, attention should be paid to acupuncture prevention in AD. (5) Finally, potential mechanisms (markers in neuroimage, biochemical, and gene) of acupuncture for AD should be studied more closely.

## 5. Conclusion

According to our findings, acupuncture therapy has been demonstrated to be effective against AD in terms of improving cognitive function, the ability of daily living, and response rate. EA + CM may be the optimal acupuncture method for improving AD cognitive function and ADL. Meanwhile, WA therapy was deemed the most effective treatment in terms of response rate. However, the overall quality of evidences was ranked as low to critically low. Therefore, well-designed and high-quality trials are expected to validate and re-evaluate scientific discoveries.

## Figures and Tables

**Table 1:** Main characteristics of included RCTs.

Study	Language	Sample size	Allocation ratio	Diagnostic criteria	Age (year)	Gender (M:F)	Course of the disease (year)	Severity	(A) Treatment group	(B) Control group I	(C) Control group II	Duration of treatment	Efficacy and safety criteria	Main results
Wang, 2021 [31]	Chinese	100	1:1	①⑥	A: 69.79 ± 6.52 B: 71.47 ± 6.32	A: 27:23 B: 26:24	A: 5.54 ± 2.25 B: 5.39 ± 2.03	NR	MA + CM	CM (AChEI, 3–6 mg/day dose of rivastigmine)	NR	90 d	1. MMSE 2. ADAS-cog 3. Response rate 4. ADL 5. AE	1. A > B 2. A > B 3. A > B 4. A > B 5. A = B
Qin, 2020 [32]	Chinese	72	1:1	③	A: 69.85 ± 5.58 B: 79.74 ± 5.62	A: 18:16 B: 17:18	A: 5.41 ± 2.22 B: 5.09 ± 2.48	NR	MA + CM	CM (AChEI, 5–10 mg/day dose of donepezil)	NR	56 d	1. MMSE 2. Response rate 3. ADL	1. A > B 2. A > B 3. A > B
Tang, 2020 [33]	Chinese	60	1:1	③⑤	A: 68.63 ± 2.63 B: 69.02 ± 2.41	A: 14:16 B: 16:14	A: 1.36 ± 0.47 B: 1.48 ± 0.49	NR	WA	MA	NR	28 d	1. MMSE 2. Response rate 3. ADL	1. A = B 2. A > B 3. A > B
Xia, 2020a [34]	Chinese	60	1:1	③	A: 61 ± 8 B: 62 ± 7	A: 17:13 B: 18:12	A: 4.3 ± 2.23 B: 4.58 ± 2.12	NR	EA + CM	CM (AChEI, 5–10 mg/day dose of donepezil)	NR	56 d	1. ADAS-cog	1. A > B
Xia, 2020b [35]	Chinese	60	1:1	③	A: 49 ± 11 B: 50 ± 12	A: 16:14 B: 12:18	A: 3.79 ± 0.27 B: 4.07 ± 0.27	NR	EA + CM	CM (AChEI, 5–10 mg/day dose of donepezil)	NR	56 d	1. MMSE 2. ADL 3. AE	1. A > B 2. A > B 3. A = B
Zhang, 2019 [36]	Chinese	92	1:1	④	A: 72.6 ± 9.2 B: 71.7 ± 8.7	A: 25:21 B: 27:19	NR	NR	FA + CM	CM (AChEI, 5–10 mg/day dose of donepezil)	NR	90 d	1. MMSE 2. Response rate 3. ADL 4. AE	1. A > B 2. A > B 3. A > B 4. A > B
Tang, 2019 [37]	Chinese	60	1:1	③⑤	A: 69 ± 1.6 B: 69.36 ± 2.10	A: 16:14 B: 15:15	A: 3.7 ± 1.57 B: 3.36 ± 1.44	NR	WA	MA	NR	28 d	1. MMSE 2. Response rate 3. ADL	1. A = B 2. A > B 3. A > B
Yuan, 2019 [38]	Chinese	44	1:1	⑥	A: 74.8 ± 3.5 B: 75.8 ± 2.8	A: 11:11 B: 12:10	NR	NR	SA + CM	CM (AChEI, 5 mg/day dose of donepezil)	NR	56 d	1. MMSE 2. Response rate	1. A = B 2. A > B
Feng, 2019 [39]	Chinese	40	1:1	③	A: 68.1 ± 4.8 B: 66.9 ± 4.2	A: 11:14 B: 12:13	A: 0.74 ± 0.06 B: 0.72 ± 0.03	NR	EA	CM (AChEI, 5 mg/day dose of donepezil)	NR	84 d	1. MMSE 2. AE	1. A = B 2. A > B
Wang, 2018 [40]	Chinese	60	1:1	①②	A: 62.23 ± 3.63 B: 61.98 ± 3.58	A: 14:16 B: 13:17	NR	NR	MA + CM	CM (AChEI, 5–10 mg/day dose of donepezil)	NR	124 d	1. MMSE 2. ADAS-cog 3. ADL	1. A > B 2. A > B 3. A > B
Jiang, 2018 [41]	Chinese	40	1:1	⑥⑦	60–80	11:9	2–12	Mild and moderate	SA	CM (AChEI, 5–10 mg/day dose of donepezil)	NR	84 d	1. MMSE 2. ADL	1. A < B 2. A < B
He, 2018 [42]	Chinese	60	1:1	①③	A: 67.53 ± 5.54 B: 68.37 ± 5.32	A: 14:16 B: 13:17	A: 1.82 ± 0.74 B: 1.94 ± 0.84	NR	WA + CM	CM (AChEI, 5 mg/day dose of donepezil)	NR	84 d	1. MMSE 2. Response rate	1. A > B 2. A > B
Feng, 2017 [43]	Chinese	40	1:1	③⑨	A: 68.10 ± 8.66 B: 68.95 ± 7.16	A: 12:8 B: 7:13	A: 4.23 ± 2.26 B: 4.23 ± 2.49	NR	EA	CM (AChEI, 5–10 mg/day dose of donepezil)	NR	84 d	1. MMSE 2. AE	1. A = B 2. A > B
Jia, 2017 [44]	English	87	43:44	①②	A: 75.11 ± 6.53 B: 74.5 ± 6.83	A: 13:30 B: 16:28	A: 3.2 ± 1.9 B: 3.0 ± 1.4	A: 24 cases of mild and 19 cases of moderate B: 23 cases of mild and 21 cases of moderate	MA	CM (AChEI, 5–10 mg/day dose of donepezil)	NR	112 d	1. ADAS-cog 2. AE	1. A = B 2. A < B
Peng, 2017 [45]	English	50	1:1	①⑩	A: 69.4 ± 5.4 B: 69.5 ± 5.3	A: 12:13 B: 12:13	A: 7.5 ± 1.8 B: 7.6 ± 1.7	NR	EA + CM	CM (AChEI, 0.2 mg/day dose of huperzine)	NR	30 d	1. MMSE 2. Response rate	1. A > B 2. A > B
Ben, 2016 [46]	English	74	1:1	①	A: 71.5 ± 4.7 B: 70.2 ± 4.6	A: 16:21 B: 17:20	A: 3.2 ± 1.9 B: 3.0 ± 1.4	A: 27 cases of mild and 10 cases of moderate B: 25 cases of mild and 12 cases of moderate	EA	CM (AChEI, 5–10 mg/day dose of donepezil)	NR	84 d	1. MMSE 2. Response rate	1. A > B 2. A = B
Lin, 2016 [47]	Chinese	90	1:1:1	①⑧	A: 73.2 ± 4.81 B: 69.7 ± 5.39 C: 71.6 ± 5.22	A: 14:16 B: 18:12 C: 17:13	A: 0.15 ± 0.02 B: 0.16 ± 0.02 C: 0.17 ± 0.02	NR	SA + CM	SA	CM (AChEI, 5 mg/day dose of donepezil)	84 d	1. MMSE 2. ADAS-cog 3. Response rate 4. ADL	1. A > B > C 2. A > B = C 3. A > B = C 4. A > B > C
Wang, 2015 [48]	Chinese	72	1:1	②	A: 72.05 ± 3.7 B: 70.31 ± 3.79	A: 16:20 B: 19:17	A: 3.33 ± 1.98 B: 2.6 ± 1.51	NR	EA	CM (AChEI, 5–10 mg/day dose of donepezil)	NR	84 d	1. MMSE 2. Response rate	1. A > B 2. A = B
Liu, 2015 [49]	Chinese	40	1:1	②⑧	A: 72.2 ± 4.8 B: 74.4 ± 4.7	A: 9:11 B: 11:9	A: 3.38 ± 1.12 B: 2.98 ± 1.07	NR	MA	CM (AChEI, 5 mg/day dose of donepezil)	NR	84 d	Response rate	A = B
Li, 2014a [50]	Chinese	40	1:1	②	NR	NR	NR	NR	SA	CM (AChEI, 5 mg/day dose of donepezil)	NR	56 d	1. MMSE 2. ADL	1. A > B 2. A > B
Wang, 2014 [51]	English	55	27:28	①	A: 70.7 ± 9.1 B: 70.3 ± 8.0	A: 13:15 B: 14:13	A: 0.48 ± 0.05 B: 0.42 ± 0.09	NR	SA + CM	CM (AChEI, 5–10 mg/day dose of donepezil)	NR	20 d	1. MMSE 2. ADAS-cog 3. Response rate	1. A > B 2. A = B 3. A > B
Ke, 2014 [52]	Chinese	64	1:1	①②⑤	A: 68.28 ± 2.54 B: 68.75 ± 3.40	A: 15:17 B: 16:16	A: 1.05 ± 0.22 B: 0.93 ± 0.23	NR	MA	CM (AChEI, 5 mg/day dose of donepezil)	NR	28 d	1. MMSE 2. Response rate 3. ADL	1. A > B 2. A = B 3. A > B
Ni, 2014 [53]	Chinese	60	1:1	①②⑤	A: 71.8 ± 5.07 B: 70.37 ± 4.96	A: 15:15 B: 14:16	A: 1.23 ± 0.4 B: 1.21 ± 0.39	NR	MA	CM (AChEI, 5 mg/day dose of donepezil)	NR	28 d	1. MMSE 2. Response rate 3. ADL	1. A > B 2. A = B 3. A > B
Zhang, 2014 [54]	Chinese	60	1:1	①②⑤	A: 70.67 ± 4.19 B: 72.43 ± 4.25	A: 13:17 B: 14:17	A: 1.02 ± 0.24 B: 1.15 ± 0.23	NR	MA	CM (AChEI, 5 mg/day dose of donepezil)	NR	28 d	1. MMSE 2. Response rate 3. ADL	1. A > B 2. A = B 3. A = B
Yang, 2014 [55]	Chinese	60	1:1	①②⑤	A: 69.97 ± 5.26 B: 70.23 ± 6.30	A: 16:14 B: 15:15	A: 1.24 ± 0.38 B: 1.24 ± 0.43	NR	MA	CM (AChEI, 5 mg/day dose of donepezil)	NR	28 d	1. MMSE 2. Response rate 3. ADL	1. A > B 2. A = B 3. A > B
Li, 2014b [56]	Chinese	60	1:1	①⑧	60–70	A: 17:13 B: 16:14	NR	A: 14 cases of mild and 16 cases of moderate B: 15 cases of mild and 15 cases of moderate	MA + CM	CM (AChEI, 5 mg/day dose of donepezil)	NR	56 d	1. MMSE 2. ADAS-cog 3. Response rate 4. ADL	1. A = B 2. A > B 3. A = B 4. A = B
Lin, 2014 [57]	Chinese	36	1:1	①②	A: 73.44 ± 3.37 B: 74.21 ± 2.68	A: 7:11 B: 8:10	A: 1.40 ± 0.62 B: 1.29 ± 0.81	36 cases of mild	MA	CM (AChEI, 5 mg/day dose of donepezil)	NR	84 d	1. MMSE 2. ADAS-cog 3. Response rate 4. ADL	1. A = B 2. A > B 3. A = B 4. A = B
Yan, 2014 [58]	Chinese	40	1:1	④	A: 60–78 B: 60–80	A: 10:10 B: 8:12	A: 0–2 B: 0–2	A: 16 cases of mild and moderate and 4 cases of severe B: 15 cases of mild and moderate and 5 cases of severe	SA	CM (AChEI, 5 mg/day dose of donepezil)	NR	84 d	MMSE	A < B
Gu, 2014 [59]	Chinese	160	1:1	①②	A: 75 ± 7 B: 72 ± 7	A: 22:50 B: 20:49	A: 1.27 ± 0.32 B: 1.34 ± 0.24	NR	MA	CM (AChEI, 5 mg/day dose of donepezil)	NR	112 d	1. MMSE 2. ADAS-cog 3. ADL	1. A > B 2. A > B 3. A > B
Sun, 2013 [60]	Chinese	70	1:1	⑧	A: 64.56 ± 9.05 B: 64.4 ± 9.12	A: 20:15 B: 14:21	A: 4.96 ± 2.31 B: 5.16 ± 2.48	NR	SA + CM	CM (AChEI, 5 mg/day dose of donepezil)	NR	32 d	1. MMSE 2. ADL	1. A > B 2. A > B
Yin, 2013 [61]	Chinese	60	1:1	①②	60–85	24:36	NR	NR	SA + CM	CM (AChEI, 5 mg/day dose of donepezil)	NR	84 d	1. MMSE 2. ADL	1. A > B 2. A > B
Zhu, 2010 [62]	Chinese	40	1:1	①	72.3 ± 6	NR	0.5–3	Mild and moderate	MA	CM (AChEI, 5 mg/day dose of donepezil)	NR	56 d	MMSE	A < B
Jiang, 2004 [63]	Chinese	44	6:5	①⑧	A: 65.1 ± 6.4 B: 64.3 ± 5.2	A: 14:10 B: 12:8	A: 3 ± 1.3 B: 3 ± 1.6	NR	MA	CM (AChEI, 0.2 mg/day dose of huperzine)	NR	56 d	1. MMSE 2. ADL	1. A = B 2. A = B
Dong, 2002 [64]	Chinese	21	11:10	①	46–80	20:12	0.25–10	NR	EA	CM (AChEI, 0.2 mg/day dose of huperzine)	NR	90 d	1. MMSE 2. ADL	1. A = B 2. A = B

*Notes*. ①: DSM, ②: NINCDS-ADRDA, ③: NIA-AA, ④: international classification of diseases-10 (ICD-10), ⑤: TCM Dementia Syndrome Classification Scale (SDSD), ⑥: guidelines for the diagnosis and treatment of dementia and cognitive impairment in China, ⑦: guiding principles of clinical research on the treatment of senile dementia with new Chinese medicine, ⑧: clinical diagnosis and curative effect evaluation standard of traditional Chinese medicine for senile dementia, ⑨: operational diagnostic criterion for AD (OCDAD), ⑩: Neuroepidemiology Branch of the National Institute of Neurological Disorders and Stroke convened an International Workshop with support from the Association Internationale pour la Recherche et l'Enseignement en Neurosciences (NINDS-AIREN), NR: not recorded, MA: manual acupuncture, EA: electroacupuncture, FA: fire acupuncture, WA: warm acupuncture, SA: scalp acupuncture, CM: conventional medicine, AChEI: acetylcholinesterase inhibitor, MMSE: the Mini-Mental State Examination, ADAS-cog: the Alzheimer's Disease Assessment Scale-Cognitive, and ADL: activities of daily living.

**Table 2:** Details of acupuncture methods according to STRICTA.

Study	Acupuncture rationale			Details of needling							Treatment regimen		Other components		Practitioner	Comparator interventions	
	1a	1b	1c	2a	2b	2c	2d	2e	2f	2g	3a	3b	4a	4b	5	6a	6b
Wang, 2021 [31]	TCM	Y	Y	13	Neiguan (PC 6), Sanyinjiao (SP 6), Shuigou (DU 26), Fengchi (GB 20), Wangu (GB 12), Yifeng (SJ 17), Jingjin and Yuye (EX-HN12)	13–60 mm	Deqi	Manual	NR	Diameter and length: NR and 75 mm Needle brand: NR	60	Frequency: 1 time per day Duration: 60 days	NR	NR	NR	Y	Y
Qin, 2020 [32]	TCM	Y	Y	6	Baihui (DU 20), Fengfu (DU 16), Fengchi (GB 20), Dazhui (DU 14), Shuigou (DU 26)	13–40 mm	Deqi	Manual	30 min	Diameter and length: NR and 40 mm Needle brand: Hwato	48	Frequency: 6 times per week Duration: 8 weeks	NR	Y	Y	Y	Y
Tang, 2020 [33]	TCM	Y	Y	15	Shenmai (BL 62), Zhaohai (KI 6), Baihui (DU 20), Sishencong (EX-HN1), Fengfu (DU 16), Taixi (KI 3), Xuanzhong (GB 39), Zusanli (ST 36), Shenshu (BL 23)	≤25 mm	Deqi	Manual	40 min	Diameter and length: 0.3 mm and 40 mm Needle brand: Andi	24	Frequency: 6 times per week Duration: 4 weeks	Y	NR	NR	NR	Y
Xia, 2020a [34]	TCM	Y	Y	2	Baihui (DU 20), Fengfu (DU 16)	15–20 mm	Deqi	Electrical	40 min	Diameter and length: 0.35 mm and 40 mm Needle brand: NR EA: KWD-808i EA apparatus	56	Frequency: 1 time per day Duration: 8 weeks	NR	Y	Y	NR	Y
Xia, 2020b [35]	TCM	Y	Y	2	Baihui (DU 20), Fengfu (DU 16)	≤40 mm	Deqi	Electrical	40 min	Diameter and length: 0.35 mm and 40 mm Needle brand: Hwato EA: Hwato EA apparatus	56	Frequency: 1 time per day Duration: 8 weeks	NR	Y	NR	Y	Y
Zhang, 2019 [36]	TCM	Y	Y	5/8	Baihui (DU 20), Pishu (BL20), Shenshu (BL 23)/Xinshu (BL 15), Zusanli (ST 36), Sishencong (EX-HN1)	5–12 mm	NR	Fire	NR	NR	12	Frequency: 1 time per week Duration: 3 months	NR	NR	NR	Y	Y
Tang, 2019 [37]	TCM	Y	Y	11	Baihui (DU 20), Fengfu (DU 16), Danzhong(RN 17), Zusanli (ST 36), Dazhu (BL 11), Shangjuxu (ST 37), Xiajuxu (ST 39)	≤40 mm	Deqi	Manual	40 min	Diameter and length: 0.3 mm and 40 mm Needle brand: Andi	24	Frequency: 6 times per week Duration: 4 weeks	NR	NR	NR	NR	NR
Yuan, 2019 [38]	TCM	Y	Y	4	Sishenzhen, Niesanzhen, Naosanzhen, Zhisanzhen	≤40 mm	NR	Manual	30 min	Diameter and length: 0.3 mm and 40 mm Needle brand: Huanqiu	40	Frequency: 5 times per week Duration: 8 weeks	NR	Y	NR	NR	NR
Feng, 2019 [39]	TCM	Y	Y	8	Baihui (DU 20), Fengfu (DU 16), Shenting (DU 24), Taiyang (EX-HN 5), Shangyintang, Dazhong (KI 4)	15–25 mm	Deqi	Electrical	30 min	Diameter and length: 0.2 mm and 25 mm Needle brand: NR EA: SDZ-V EA apparatus	36	Frequency: 3 times per week Duration: 12 weeks	NR	Y	NR	Y	Y
Wang, 2018 [40]	TCM	Y	Y	8	Danzhong(RN 17), Zhongwan (RN 12), Qihai (RN 6), Zusanli (ST 36), Waiguan (SJ 5), Xuehai (SP 10)	≤40 mm	NR	Manual	NR	Diameter and length: NR and 40 mm Needle brand: Hwato	126	Frequency: 1 time per day Duration: 18 weeks	NR	NR	NR	Y	Y
Jiang, 2018 [41]	TCM	Y	Y	3	Naohu (DU 17), Naokong (GB 19), Shenting (DU 24), Benshen (GB 13), 3 points (2 cuns straight up the erjian, 1 cun front, and 1 cun back)	≤25 mm	NR	Manual	30 min	Diameter and length: 0.3 mm and 25 mm Needle brand: Hwato	65–66	Frequency: 7 times 9 days Duration: 12 weeks	NR	Y	NR	NR	Y
He, 2018 [42]	TCM	Y	Y	10	Baihui (DU 20), Dazhui (DU 14), Zhiyang (DU 9), Mingmen (DU 4), Shenshu (BL 23), Taixi (KI 3), Xuanzhong (GB 39)	≤30 mm	Deqi	Manual	30 min	Diameter and length: 0.25 mm and 40 mm Needle brand: Jiajian	72	Frequency: 6 times per weeks Duration: 12 weeks	NR	Y	NR	Y	Y
Feng, 2017 [43]	TCM	Y	Y	8	Baihui (DU 20), Sshenting (DU 24), Taiyang (EX-HN 5), Shangyintang, Dazhong (KI 4), Fengfu (DU 16)	15–25 mm	Deqi	Electrical	30 min	Diameter and length: 0.2 mm and 25 mm Needle brand: Hwato EA: SDZ-V EA apparatus	36	Frequency: 3 times per week Duration: 12 weeks	NR	Y	NR	Y	Y
Jia, 2017 [44]	TCM	Y	Y	9	Danzhong (RN 17), Zhongwan (RN 12), Qihai (RN 6), Zusanli (ST 36), Waiguan (SJ 5), Xuehai (SP 10)	15–25 mm	Deqi	Manual	30 min	Diameter and length: 0.3 mm and 40 mm Needle brand: Hwato	36	Frequency: 3 times per week Duration: 12 weeks	NR	Y	Y	Y	Y
Peng, 2017 [45]	TCM	Y	Y	7	Shenting (DU 24), Baihui (DU 20), Dazhui (DU14), Fengfu (DU 16), Mingmen (DU 4), Yongquan (KI 1)	≤40 mm	Deqi	Electrical	25 min	Diameter and length: 0.25 mm and (25–40) mm Needle brand: Hwato EA: G6805-II EA apparatus	30	Frequency: 1 time per day Duration: 3 months	NR	Y	NR	Y	Y
Ben, 2016 [46]	TCM	Y	Y	4	Zusanli (ST 36), Fenglong (ST 40)	25–60 mm	Deqi	Electrical	30 min	Diameter and length: 0.3 mm and 25–60 mm Needle brand: NR EA: G91-D EA apparatus	72	Frequency: 6 times per week Duration: 12 weeks	NR	Y	NR	NR	Y
Lin, 2016 [47]	TCM	Y	Y	13	Naohu (DU 17), Naokong (GB 19), Shenting (DU 24), Benshen (GB 13), 3 points (2 cuns straight up the erjian, 1 cun front, and 1 cun back)	≤40 mm	Deqi	Manual	30 min	Diameter and length: 0.3 mm and 40 mm Needle brand: Huanqiu	60	Frequency: 5 times per week Duration: 12 weeks	NR	Y	NR	Y	Y
Wang, 2015 [48]	TCM	Y	Y	2	Baihui (DU 20), Fengfu (DU 16)	13–25 mm	Deqi	Electrical	30 min	Diameter and length: 0.3 mm and 25 mm Needle brand: Hwato EA: G6805 EA apparatus	72	Frequency: 6 times per week Duration: 12 weeks	NR	Y	NR	NR	Y
Liu, 2015 [49]	TCM	Y	Y	2	Baihui (DU 20), Dazhui (DU 14)	13 mm	NR	Manual	40 min	Diameter and length: NR and 30–40 mm Needle brand: NR	84	Frequency: 1 time per week Duration: 12 weeks	NR	Y	NR	NR	Y
Li, 2014a [50]	WM	Y	Y	15	Parietal area, preparietal area, frontal area	≤40 mm	Deqi	Manual	30 min	Diameter and length: 0.35 mm and 40 mm Needle brand: NR	56	Frequency: 1 time per week Duration: 8 weeks	NR	Y	NR	NR	Y
Wang, 2014 [51]	TCM	Y	Y	4	Coronal suture, sagittal suture, lambdoidal suture, frontotemporal sutures	25–35 mm	NR	Manual	30 min	Diameter and length: 0.3 mm and 40 mm Needle brand: Hwato	20	Frequency: 1 time per week Duration: 20 days	NR	Y	NR	Y	Y
Ke, 2014 [52]	TCM	Y	Y	8	Yintang (DU 29), Baihui (DU 20), Sishencong (EX-HN1), Xuanzhong (GB 39)	13–50 mm	NR	Manual	40 min	Diameter and length: 0.35 mm and 40 mm Needle brand: Hwato	24	Frequency: 6 times per week Duration: 4 weeks	NR	Y	NR	Y	Y
Ni, 2014 [53]	TCM	Y	Y	8	Baihui (DU 20), Sishencong (EX-HN1), Yintang (DU 29), Xuanzhong (GB 39)	2.5–20 mm	NR	Manual	40 min	Diameter and length: 0.35 mm and 40 mm Needle brand: Hwato	24	Frequency: 6 times per week Duration: 4 weeks	NR	Y	NR	Y	Y
Zhang, 2014 [54]	TCM	Y	Y	8	Baihui (DU 20), Sishencong (EX-HN1), Yintang (DU 29), Xuanzhong (GB 39)	7.5–50 mm	NR	Manual	40 min	Diameter and length: 0.35 mm and 40 mm Needle brand: Hwato	24	Frequency: 6 times per week Duration: 4 weeks	NR	Y	NR	Y	Y
Yang, 2014 [55]	TCM	Y	Y	8	Baihui (DU 20), Sishencong (EX-HN1), Yintang (DU 29), Xuanzhong (GB 39)	7.5–50 mm	NR	Manual	40 min	Diameter and length: 0.35 mm and 40 mm Needle brand: Hwato	24	Frequency: 6 times per week Duration: 4 weeks	NR	Y	NR	Y	Y
Li, 2014b [56]	TCM	Y	Y	10/14	Baihui (DU 20), Dazhui (DU 14), Dazhu (BL 11), Feishu (BL 13), Ganshu (BL 18), Pishu (BL20), Xinshu (BL 15), Shenshu (BL 23)/Baihui (DU 20), Zhongwan (RN 12), Tianshu (ST 25), Guanyuan (RN 4), zusanli (ST 36), Xiajuxu (ST 39)	13–32.5 mm	Deqi	Manual	30 min	Diameter and length: 0.35 mm and 25/40 mm Needle brand: Hwato	40	Frequency: 5 times per week Duration: 8 weeks	Y	Y	NR	Y	Y
Lin, 2014 [57]	TCM	Y	Y	8	Baihui (DU 20), Sishencong (EX-HN1), Neiguan (PC 6), Sanyinjiao (SP 6)	40–50 mm	Deqi	Manual	30 min	Diameter and length: 0.28–0.3 mm and 40–50 mm Needle brand: Hwato	72	Frequency: 6 times per week Duration: 12 weeks	NR	Y	NR	Y	Y
Yan, 2014 [58]	TCM	Y	Y	11	Shenting (DU 24), Benshen (GB 13), Sishencong (EX-HN1), Shenmen (HT 7), Taixi (KI 3)	13–40 mm	Deqi	Manual	20 min	Diameter and length: 0.3–0.32 mm and 13–25 mm Needle brand: Hwato	60	Frequency: 5 times per week Duration: 12 weeks	NR	NR	NR	NR	Y
Gu, 2014 [59]	TCM	Y	Y	14	Shenting (DU 24), Baihui (DU 20), Fengchi (GB 20), Wangu (GB 12), Danzhong (RN 17), Zhongwan (RN 12), Qihai (RN 6), Xuehai (SP 10), Zusanli (ST 36)	13–40 mm	Deqi	Manual	30 min	Diameter and length: 0.3 mm and 40–50 mm Needle brand: Hwato	96	Frequency: 6 times per week Duration: 16 weeks	NR	Y	NR	Y	Y
Sun, 2013 [60]	TCM	Y	Y	4	4 points (1.5 cuns far from the Baihui (DU 20))	NR	NR	Manual	30 min	NR	32	Frequency: 1 time per week Duration: 32 days	NR	Y	NR	NR	Y
Yin, 2013 [61]	TCM	Y	Y	4	Parietemporal anterior oblique line, parietemporal posterior oblique line	30–45 mm	Deqi	Manual	45 min	Diameter and length: 0.3 mm and 40–50 mm Needle brand: Hwato	84	Frequency: 1 time per week Duration: 12 weeks	NR	Y	NR	Y	Y
Zhu, 2010 [62]	TCM	Y	Y	7	Baihui (DU 20), Shenshu (BL 23), Xuehai (SP 10), Geshu (BL 17)	13–40 mm	NR	Manual	30 min	Diameter and length: 0.38 mm and 10–75 mm Needle brand: Hwato	56	Frequency: 1 time per week Duration: 8 weeks	NR	Y	NR	NR	Y
Jiang, 2004 [63]	TCM	Y	Y	7–8	Baihui (DU 20), Shenshu (BL 23), Shenmen (HT 7), Neiguan (PC 6)/Sishencong (EX-HN1), Fengchi (GB 20), Taixi (KI 3), Zusanli (ST 36)	≤40 mm	NR	Manual	30 min	Diameter and length: 0.35 mm and 40 mm Needle brand: NR	40	Frequency: 5 times per week Duration: 8 weeks	NR	NR	NR	NR	Y
Dong, 2002 [64]	TCM	Y	Y	10–14	Baihui (DU 20), Dazhui (DU 14), Shenshu (BL 23), Shenmen (HT 7), Neiguan (PC 6), Sanyinjiao (SP 6)/Sishencong (EX-HN1), Fengchi (GB 20), Taixi (KI 3), Zusanli (ST 36), Fenglong (ST 40), Taichong (LR 3)	NR	Deqi	Electrical	40 min	NR	60	Frequency: 5 times per week Duration: 12 weeks	NR	NR	NR	NR	Y

*Notes*. 1a: style of acupuncture, 1b: reasoning for treatment provided, 1c: extent to which treatment was varied, 2a: number of needle insertions per subject per session, 2b: names of points used, 2c: depth of insertion, 2d: response sought, 2e: needle stimulation, 2f: needle retention time, 2g: needle type, 3a: number of treatment sessions, 3b: frequency and duration of treatment sessions, 4a: details of other interventions administered to the acupuncture group, 4b: setting and context of treatment, 5: description of participating acupuncturists, 6a: rationale for the control or comparator, 6b: precise description of the control or comparator, NR: not recorded, and Y: yes.

**Table 3:** Pairwise meta-analysis of improvement of MMSE.

Comparison		Number	WMD (95% CI)	*I* ^2^	*p*
EA + CM	CM	2	5.56 (2.10, 9.03)^*∗*^	72.4%	0.057
MA + CM	CM	5	2.43 (0.78, 4.07)^*∗*^	93.8%	<0.00001
SA + CM	CM	4	2.68 (–1.10, 6.46)	90.5%	<0.00001
WA + CM	CM	1	1.63 (–0.38, 3.64)	—	—
FA + CM	CM	1	4.14 (3.10, 5.18)^*∗*^	—	—
EA	CM	5	1.52 (–0.14, 3.18)	85.2%	<0.00001
MA	CM	9	0.72 (-0.46, 1.90)	89.3%	<0.00001
SA	CM	3	3.88 (–2.89, 10.65)	95.5%	<0.00001
WA	MA	2	0.51 (0.02, 1.00)^*∗*^	0%	0.817
SA + CM	SA	1	0.20 (–3.00, 3.40)	—	—

*Notes.* ^*∗*^Significant difference, MA: manual acupuncture, EA: electroacupuncture, FA: fire acupuncture, WA: warm acupuncture, SA: scalp acupuncture, CM: conventional medicine, and MMSE: the Mini-Mental State Examination.

**Table 4:** Pairwise meta-analysis of reduction of ADAS-cog.

Comparison		Number	WMD (95% CI)	*I* ^2^	*p*
EA + CM	CM	1	4.32 (1.55, 7.09)^*∗*^	—	—
MA + CM	CM	3	2.46 (1.12, 3.80)^*∗*^	67.0%	0.049
SA + CM	CM	2	4.43 (–0.06, 8.92)	76.7%	0.038
MA	CM	3	3.11 (1.74, 4.47)^*∗*^	31.7%	0.231
SA	CM	1	2.00 (–0.30, 4.30)	—	—
SA + CM	SA	1	4.50 (2.18, 6.82)^*∗*^	—	—

*Notes.* ^*∗*^Significant difference, MA: manual acupuncture, EA: electroacupuncture, SA: scalp acupuncture, CM: conventional medicine, and ADAS-cog: the Alzheimer's Disease Assessment Scale-Cognitive.

**Table 5:** Pairwise meta-analysis of response rate.

Comparison		Number	RR (95% CI)	*I* ^2^	*p*
EA + CM	CM	1	1.08 (0.69, 1.71)	—	—
MA + CM	CM	3	1.18 (0.92, 1.52)	1.7%	0.362
SA + CM	CM	3	1.20 (0.91, 1.56)	0%	0.937
WA + CM	CM	1	1.20 (0.76, 1.88)	—	—
FA + CM	CM	1	1.17 (0.81, 1.69)	—	—
EA	CM	2	1.09 (0.83, 1.44)	0%	0.837
MA	CM	6	1.25 (1.02, 1.54)^*∗*^	0%	0.986
SA	CM	1	1.19 (0.74, 1.90)	—	—
WA	MA	2	1.29 (0.90, 1.84)	0%	0.855
SA + CM	SA	1	1.07 (0.71, 1.60)	—	—

*Notes.* ^*∗*^Significant difference, MA: manual acupuncture, EA: electroacupuncture, FA: fire acupuncture, WA: warm acupuncture, SA: scalp acupuncture, and CM: conventional medicine.

**Table 6:** Pairwise meta-analysis of improvement of ADL.

Comparison		Number	WMD (95% CI)	*I* ^2^	*p*
EA + CM	CM	1	8.01 (3.23, 12.79)^*∗*^	—	—
MA + CM	CM	5	3.90 (2.29, 5.52)^*∗*^	66.6%	0.018
SA + CM	CM	2	4.49 (–4.17, 13.15)	96.8%	<0.00001
FA + CM	CM	1	1.63 (0.11, 3.15)^*∗*^	—	—
EA	CM	1	0.48 (–4.30, 3.34)	—	—
MA	CM	7	1.92 (1.31, 2.52)^*∗*^	0%	0.709
SA	CM	3	3.17 (1.49, 4.85)^*∗*^	0%	0.658
WA	MA	2	1.82 (1.15, 2.49)^*∗*^	0%	0.413
SA + CM	SA	1	4.90 (2.06, 7.74)^*∗*^	—	—

*Notes.* ^*∗*^Significant difference, MA: manual acupuncture, EA: electroacupuncture, FA: fire acupuncture, WA: warm acupuncture, SA: scalp acupuncture, CM: conventional medicine, and ADL: activities of daily living.

**Table 7:** The results of network meta-analysis of improvement of MMSE.

EA + CM									

1.38 (−5.09, 7.76)	FA + CM								
3.27 (−1.35, 7.83)	1.92 (−3.64, 7.38)	SA + CM							
4.25 (−0.72, 9.02)	2.90 (−3.01, 8.53)	0.96 (−2.50, 4.27)	SA						
3.94 (−0.71, 8.48)	2.58 (−3.03, 8.07)	0.69 (−2.63, 4.00)	−0.30 (−3.80, 3.32)	EA					
3.02 (−1.73, 7.68)	1.62 (−4.10, 7.22)	−0.27 (−3.71, 3.17)	−1.25 (−4.93, 2.67)	−0.94 (−4.40, 2.51)	MA + CM				
4.43 (0.04, 8.70)^*∗*^	3.03 (−2.47, 8.28)	1.11 (−1.85, 4.12)	0.14 (−3.11, 3.59)	0.43 (−2.44, 3.40)	1.36 (−1.79, 4.54)	MA			
4.00 (−1.77, 9.60)	2.64 (−3.98, 8.94)	0.69 (−4.05, 5.44)	−0.29 (−5.12, 4.77)	−0.02 (−4.71, 4.64)	0.96 (−3.86, 5.77)	−0.41 (−4.03, 3.21)	WA		
3.85 (−2.82, 10.46)	2.47 (−4.81, 9.91)	0.53 (−5.25, 6.55)	−0.42 (−6.46, 5.83)	−0.13 (−5.99, 5.78)	0.87 (−5.12, 6.92)	−0.57 (−6.19, 5.25)	−0.09 (−6.89, 6.73)	WA + CM	
5.49 (1.51, 9.41)^*∗*^	4.14 (−1.07, 9.12)	2.21 (−0.16, 4.62)	1.26 (−1.42, 4.10)	1.53 (−0.74, 3.86)	2.46 (−0.06, 5.07)	1.09 (−0.71, 2.90)	1.53 (−2.57, 5.65)	1.66 (−3.83, 7.03)	CM

*Notes.* ^*∗*^Significant difference, MA: manual acupuncture, EA: electronic acupuncture, WA: warm acupuncture, FA: fire acupuncture, SA: scalp acupuncture, and CM: conventional medicine.

**Table 8:** The results of network meta-analysis of reduction of ADAS-cog.

EA + CM					

−0.41 (−6.45, 6.11)	SA + CM				
1.78 (−4.14, 7.46)	2.20 (−2.72, 6.45)	MA + CM			
1.04 (−4.93, 6.75)	1.51 (−3.81, 5.77)	−0.72 (−4.86, 3.24)	MA		
3.15 (−3.54, 10.23)	3.62 (−1.14, 8.13)	1.38 (−3.98, 7.02)	2.11 (−3.05, 7.87)	SA	
4.30 (−0.87, 9.37)	4.70 (0.76, 8.20)^*∗*^	2.49 (−0.30, 5.43)	3.21 (0.62, 6.28)^*∗*^	1.12 (−3.65, 5.62)	CM

*Notes.* ^*∗*^Significant difference, MA: manual acupuncture, EA: electronic acupuncture, SA: scalp acupuncture, and CM: conventional medicine.

**Table 9:** The results of network meta-analysis of response rate.

WA									

4.00 (0.73, 20.25)	WA + CM								
7.24 (0.94, 39.36)	1.74 (0.20, 12.51)	EA + CM							
5.44 (1.30, 16.01)^*∗*^	1.34 (0.24, 6.60)	0.79 (0.14, 4.30)	MA + CM						
3.03 (0.59, 10.32)	0.75 (0.13, 6.29)	0.44 (0.06, 2.86)	0.59 (0.15, 1.78)	SA + CM					
5.70 (1.05, 23.05)^*∗*^	1.57 (0.23, 8.41)	0.84 (0.13, 6.42)	1.11 (0.30, 4.05)	1.98 (0.48, 8.53)	FA + CM				
3.86 (1.45, 9.02)^*∗*^	0.92 (0.22, 6.89)	0.55 (0.12, 3.08)	0.71 (0.30, 1.87)	1.20 (0.47, 4.17)	0.64 (0.19, 2.41)	MA			
7.21 (1.77, 33.77)^*∗*^	1.83 (0.32, 12.15)	1.17 (0.17, 6.66)	1.46 (0.48, 4.75)	2.28 (0.79, 10.79)	1.29 (0.32, 5.61)	1.80 (0.69, 6.87)	EA		
6.53 (1.24, 28.38)^*∗*^	1.57 (0.25, 18.37)	0.93 (0.14, 6.68)	1.26 (0.30, 5.18)	2.41 (0.58, 8.74)	1.12 (0.21, 5.93)	1.77 (0.47, 6.14)	0.98 (0.16, 4.07)	SA	
17.32 (5.47, 42.46)^*∗*^	4.23 (1.04, 22.97)^*∗*^	2.41 (0.55, 12.54)	3.13 (1.62, 6.96)^*∗*^	5.53 (2.40, 16.59)^*∗*^	2.86 (0.99, 9.10)	4.44 (2.52, 7.63)^*∗*^	2.26 (0.80, 5.15)	2.49 (0.81, 8.31)	CM

*Notes.* ^*∗*^Significant difference, MA: manual acupuncture, EA: electronic acupuncture, WA: warm acupuncture, FA: fire acupuncture, SA: scalp acupuncture, and CM: conventional medicine.

**Table 10:** The results of network meta-analysis of improvement of ADL.

EA + CM								

4.78 (−2.25, 11.71)	SA + CM							
3.69 (−3.37, 10.55)	−1.15 (−4.78, 2.55)	MA + CM						
6.27 (−1.48, 14.18)	1.52 (−3.62, 6.90)	2.66 (−2.45, 7.88)	FA + CM					
5.98 (−0.65, 12.69)	1.18 (−1.94, 4.51)	2.31 (−0.74, 5.49)	−0.31 (−5.18, 4.55)	MA				
8.42 (−0.25, 16.83)	3.61 (−2.60, 9.91)	4.71 (−1.36, 10.76)	2.06 (−5.18, 9.25)	2.41 (−3.71, 8.34)	EA			
7.94 (0.86, 15.02)^*∗*^	3.17 (−0.33, 6.73)	4.30 (0.39, 8.09)^*∗*^	1.61 (−3.98, 6.94)	1.96 (−1.50, 5.28)	−0.49 (−6.86, 5.87)	SA		
3.93 (−3.43, 11.41)	−0.84 (−5.37, 3.62)	0.31 (−4.14, 4.72)	−2.34 (−8.18, 3.43)	−2.02 (−5.24, 1.14)	−4.43 (−11.05, 2.24)	−3.99 (−8.51, 0.62)	WA	
7.94 (1.53, 14.49)^*∗*^	3.14 (0.54, 5.90)^*∗*^	4.26 (1.81, 6.83)^*∗*^	1.62 (−2.99, 6.12)	1.94 (0.07, 3.76)^*∗*^	−0.46 (−6.13, 5.27)	−0.01 (−2.81, 2.91)	3.97 (0.41, 7.63)^*∗*^	CM

*Notes.* ^∗^Significant difference, MA: manual acupuncture, EA: electronic acupuncture, WA: warm acupuncture, FA: fire acupuncture, SA: scalp acupuncture, and CM: conventional medicine.

**Table 11:** Adverse events in included RCTs.

Interventions	Study	Number of adverse events	Details of adverse events
MA + CM	Wang, 2021 [31]	5	1 case of nausea, 1 case of emesis, 2 cases of diarrhea, 1 case of cough
FA + CM	Zhang, 2019 [36]	2	2 cases of nausea and emesis
EA + CM	Xia, 2020b [35]	2	1 case of nausea, 1 case of dizziness
EA	Feng, 2019 [39]	3	2 cases of pain, 1 case of local hematoma
	Feng, 2017 [43]	3	2 cases of pain, 1 case of local hematoma
MA	Jia, 2017 [44]	5	4 cases of punctate hemorrhage, 1 case of local blood stasis
	Wang, 2021 [31]	4	2 cases of nausea, 1 cases of diarrhea, 1 case of cough
CM	Xia, 2020b [35]	1	1 case of nausea
	Zhang, 2019 [36]	8	2 cases of diarrhea, 5 cases of nausea and emesis, 1 case of insomnia
	Jia, 2017 [44]	7	7 cases of dizziness, nausea, loss of appetite, diarrhea, constipation, fatigue, restlessness

*Notes.* MA: manual acupuncture, EA: electronic acupuncture, FA: fire acupuncture, and CM: conventional medicine.

## Data Availability

No additional data are available.

## Supplementary Materials

Supplementary Materials Supplementary materials for this article are shown in Table S1–S8. Table S1. Search strategies of each database. Table S2. Full-text articles excluded with reasons. Table S3. (a) Node-splitting test result of improvement of MMSE and (b) node-splitting test result of improvement of ADL. Table S4. (a) The PSRF value of improvement of MMSE, (b) the PSRF value of reduction of ADAS-cog, (c) the PSRF value of response rate, and (d) the PSRF value of improvement of ADL. Table S5. Quality of evidence of improvement of MMSE in network meta-analysis. Table S6. Quality of evidence of reduction of ADAS-cog in network meta-analysis. Table S7. Quality of evidence of response rate in network meta-analysis. Table S8. Quality of evidence of improvement of ADL in network meta-analysis. Appendix 1. PRISMA-NMA checklist of items to include when reporting a systematic review involving a network meta-analysis.
