# Supplementary material for: The Comparative Efficacy of Multiple Acupuncture for Alzheimer's Disease: A Bayesian Network Meta-Analysis
Source: Evid Based Complement Alternat Med. 2022 May 17;2022:3288948. doi: 10.1155/2022/3288948 (PMC9129975; doi:10.1155/2022/3288948)
Supplement: Supplementary Materials — for this article are shown in Table S1–S8. Table S1. Search strategies of each database. Table S2. Full-text articles excluded with reasons. Table S3. (a) Node-splitting test result of improvement of MMSE and (b) node-splitting test result of improvement of ADL. Table S4. (a) The PSRF value of improvement of MMSE, (b) the PSRF value of reduction of ADAS-cog, (c) the PSRF value of response rate, and (d) the PSRF value of improvement of ADL. Table S5. Quality of evidence of improvement of MMSE in network meta-analysis. Table S6. Quality of evidence of reduction of ADAS-cog in network meta-analysis. Table S7. Quality of evidence of response rate in network meta-analysis. Table S8. Quality of evidence of improvement of ADL in network meta-analysis. Appendix 1. PRISMA-NMA checklist of items to include when reporting a systematic review involving a network meta-analysis. [file 3288948.f1.zip › 3288948.f1/Supplemental material.doc]

**Supplemental material**

**Table S1. Search strategies of each database**

**PubMed**

#1 "Alzheimer Disease"[Mesh]

#2 "Alzheimer Disease" [Title/Abstract] OR "Alzheimer's Disease" [Title/Abstract] OR "Alzheimer Dementia" [Title/Abstract] OR "Alzheimer*" [Title/Abstract] OR "Alzheimer Type Dementia" [Title/Abstract] OR "Senile Dementia" [Title/Abstract] OR "AD" [Title/Abstract] OR "ATD" [Title/Abstract]

#3 #1 OR #2

#4 "Acupuncture therapy"[Mesh] OR "Moxibustion"[Mesh]

#5 "acupuncture therapy"[Title/Abstract] OR "acupuncture"[Title/Abstract] OR "acupuncture-moxibustion"[Title/Abstract] OR "meridian*"[Title/Abstract] OR "acupoint*"[Title/Abstract] OR "warm needling"[Title/Abstract] OR "warm acupuncture"[Title/Abstract] OR "acupuncture plus moxibustion"[Title/Abstract] OR "moxibustion"[Title/Abstract] OR "electronic acupuncture"[Title/Abstract] OR "electro-acupuncture"[Title/Abstract] OR "electroacupuncture"[Title/Abstract] OR "fire acupuncture"[Title/Abstract] OR "auricular needle"[Title/Abstract] OR "scalp needle"[Title/Abstract] OR "abdominal needle"[Title/Abstract] OR "wrist ankle needle"[Title/Abstract] OR "triple puncture"[Title/Abstract] OR "dry needle"[Title/Abstract] OR "needle"[Title/Abstract] OR "body acupuncture"[Title/Abstract] OR "manual-acupuncture"[Title/Abstract]

#6 #4 OR #5

#7 "clinical trials, randomized"[Mesh Terms] OR "controlled clinical trials, randomized"[Mesh Terms] OR "clinical trials as topic"[MeSH Terms] OR "random allocation"[MeSH Terms] OR "therapeutic use"[MeSH Subheading]

#8 ("clinical"[Title/Abstract] AND "trial"[Title/Abstract]) OR "clinical trial"[Publication Type] OR "random*"[Title/Abstract]

#9 #7 OR #8

#10 #3 AND #6 AND #9

**EMBASE**

#1 'alzheimer disease'/exp/mj

#2 'alzheimer disease':ti,ab,kw OR 'alzheimers disease':ti,ab,kw OR 'alzheimer dementia':ti,ab,kw OR alzheimer:ti,ab,kw OR 'alzheimer type dementia':ti,ab,kw OR 'senile dementia':ti,ab,kw OR ad:ti,ab,kw OR atd:ti,ab,kw

#3 #1 OR #2

#4 'acupuncture therapy'/exp/mj OR 'moxibustion'/exp/mj

#5 'acupuncture therapy':ti,ab,kw OR acupuncture:ti,ab,kw OR 'acupuncture moxibustion':ti,ab,kw OR meridian*:ti,ab,kw OR acupoint*:ti,ab,kw OR 'warm needling':ti,ab,kw OR 'warm acupuncture':ti,ab,kw OR 'acupuncture plus moxibustion':ti,ab,kw OR moxibustion:ti,ab,kw OR 'electronic acupuncture':ti,ab,kw OR 'electro acupuncture':ti,ab,kw OR electroacupuncture:ti,ab,kw OR 'fire acupuncture':ti,ab,kw OR 'auricular needle':ti,ab,kw OR 'scalp needle':ti,ab,kw OR 'abdominal needle':ti,ab,kw OR 'wrist ankle needle':ti,ab,kw OR 'triple puncture':ti,ab,kw OR 'dry needle':ti,ab,kw OR needle:ti,ab,kw OR 'body acupuncture':ti,ab,kw OR 'manual acupuncture':ti,ab,kw

#6 #4 OR #5

#7 'clinical trials, randomized'/exp/mj OR 'controlled clinical trials, randomized'/exp/mj OR 'clinical trials as topic'/exp/mj OR 'random allocation'/exp/mj

#8 clinical:ti,ab,kw OR trial:ti,ab,kw OR random*:ti,ab,kw

#9 #7 OR #8

#10 #3 AND #6 AND #9

**Web of science**

TS=(‘Alzheimer Disease’ OR ‘Alzheimer's Disease’ OR ‘Alzheimer Dementia’ OR ‘Alzheimer*’ OR ‘Alzheimer Type Dementia’ OR ‘Senile Dementia’ OR ‘AD’ OR ‘ATD’) AND TS=(‘acupuncture therapy’ OR ‘acupuncture’ OR ‘acupuncture-moxibustion’ OR ‘meridian*’ OR ‘acupoint*’ OR ‘warm needling’ OR ‘warm acupuncture’ OR ‘acupuncture plus moxibustion’ OR ‘moxibustion’ OR ‘electronic acupuncture’ OR ‘electro-acupuncture’ OR ‘electroacupuncture’ OR ‘fire acupuncture’ OR ‘auricular needle’ OR ‘scalp needle’ OR ‘abdominal needle’ OR ‘wrist ankle needle’ OR ‘triple puncture’ OR ‘dry needle’ OR ‘needle’ OR ‘body acupuncture’ OR ‘manual-acupuncture’) AND TS=(‘random*’ OR ‘clinical’ OR ‘trial’ )

**Cochrane Library**

#1 Mesh descriptor: [Alzheimer Disease] explode all trees;

#2 alzheimer disease:ti,ab,kw OR alzheimers disease:ti,ab,kw OR alzheimer dementia:ti,ab,kw OR alzheimer:ti,ab,kw OR alzheimer type dementia:ti,ab,kw OR senile dementia:ti,ab,kw OR ad:ti,ab,kw OR atd:ti,ab,kw

#3 #1 OR #2

#4 Mesh descriptor: [acupuncture therapy] or [moxibustion] explode all trees;

#5 acupuncture therapy:ti,ab,kw OR acupuncture:ti,ab,kw OR acupuncture moxibustion:ti,ab,kw OR meridian*:ti,ab,kw OR acupoint*:ti,ab,kw OR warm needling:ti,ab,kw OR warm acupuncture:ti,ab,kw OR acupuncture plus moxibustion:ti,ab,kw OR moxibustion:ti,ab,kw OR electronic acupuncture:ti,ab,kw OR electro acupuncture:ti,ab,kw OR electroacupuncture:ti,ab,kw OR fire acupuncture:ti,ab,kw OR auricular needle:ti,ab,kw OR scalp needle:ti,ab,kw OR abdominal needle:ti,ab,kw OR wrist ankle needle:ti,ab,kw OR triple puncture:ti,ab,kw OR dry needle:ti,ab,kw OR needle:ti,ab,kw OR body acupuncture:ti,ab,kw OR manual acupuncture:ti,ab,kw

#6 #4 OR #5

#7 Mesh descriptor: [clinical trials, randomized] or [controlled clinical trials, randomized] or [clinical trials as topic] or [random allocation] explode all trees;

#8 clinical:ti,ab,kw OR trial:ti,ab,kw OR random*:ti,ab,kw

#9 #7 OR #8

#10 #3 AND #6 AND #9

**CNKI**

TKA=(‘针刺’+‘针灸’+‘体针’+‘手针’+‘电针’+‘温针’+‘头皮针’+‘头针’+‘耳针’+‘穴位’+‘火针’+‘腹针’+‘浮针’+‘三棱针’+‘九针’+‘经络’+‘经皮电刺激’+‘眼针’+‘舌针’+‘腕踝针’+‘刃针’+‘针刀’+‘刺络’+‘干针’+‘灸’) AND TKA=(‘阿尔茨海默’+‘老年痴呆’+‘阿尔兹海默’+‘老年性痴呆’+‘呆症’) AND TKA=(‘随机’+‘对照’)

**WF**

主题:(阿尔茨海默 OR 阿尔兹海默 OR 老年痴呆 OR 老年性痴呆 OR 呆症) and 主题:(针刺 OR 针灸 OR 体针 OR 手针 OR 电针 OR 温针 OR 头皮针 OR 头针 OR 耳针 OR 穴位 OR 火针 OR 腹针 OR 浮针 OR 三棱针 OR 九针 OR 经络 OR 经皮电刺激 OR 眼针 OR 舌针 OR 腕踝针 OR 刃针 OR 针刀 OR 刺络 OR 干针 OR 灸) and 摘要:(随机 OR 对照)

**Chongqing VIP**

**M=**(阿尔茨海默 OR 阿尔兹海默 OR 老年痴呆 OR 老年性痴呆 OR 呆症) and **M=**(针刺 OR 针灸 OR 体针 OR 手针 OR 电针 OR 温针 OR 头皮针 OR 头针 OR 耳针 OR 穴位 OR 火针 OR 腹针 OR 浮针 OR 三棱针 OR 九针 OR 经络 OR 经皮电刺激 OR 眼针 OR 舌针 OR 腕踝针 OR 刃针 OR 针刀 OR 刺络 OR 干针 OR 灸) and **R=**(随机 OR 对照)

**CBM**

1	"阿尔茨海默病" [加权:扩展]

2	"阿尔茨海默"[常用字段:智能] OR "阿尔兹海默"[常用字段:智能] OR "老年痴呆"[常用字段:智能] OR "老年性痴呆"[常用字段:智能] OR "呆症"[常用字段:智能]

3	1 OR 2

4	"针刺疗法"[加权:扩展] OR "针刺"[加权:扩展] OR "针灸疗法"[加权:扩展]

5	"针刺"[常用字段:智能] OR "针灸"[常用字段:智能] OR "体针"[常用字段:智能] OR "手针"[常用字段:智能] OR "电针"[常用字段:智能] OR "温针"[常用字段:智能] OR "头皮针"[常用字段:智能] OR "头针"[常用字段:智能] OR "耳针"[常用字段:智能] OR "穴位"[常用字段:智能] OR "火针"[常用字段:智能] OR "腹针"[常用字段:智能] OR "浮针"[常用字段:智能] OR "三棱针"[常用字段:智能] OR "九针"[常用字段:智能] OR "经络"[常用字段:智能] OR "经皮穴位电刺激"[常用字段:智能] OR "眼针"[常用字段:智能] OR "舌针"[常用字段:智能] OR "腕踝针"[常用字段:智能] OR "刃针"[常用字段:智能] OR "针刀"[常用字段:智能] OR "刺络"[常用字段:智能] OR "干针"[常用字段:智能] OR "灸"[常用字段:智能]

6	4 OR 5

7	"随机对照试验"[不加权:扩展]

8	"随机"[常用字段:智能] OR "对照"[常用字段:智能]

9	7 OR 8

10	3 AND 6 AND 9

**Table S2. Full-text articles excluded with reasons**

| Full-text articles excluded | Reasons |
| --- | --- |
| Liang et al, 2021^1^ | Non-RCT |
| Li et al, 2020^2^ | Non-RCT |
| Yang et al, 2019^3^ | Non-RCT |
| Tony 2019^4^ | Non-RCT |
| Chen et al, 2014^5^ | Non-RCT |
| Chen et al, 2015^6^ | Non-RCT |
| Yuan et al, 2013^7^ | Non-RCT |
| Ying 2006^8^ | Non-RCT |
| Du et al, 2019^9^ | Ineligible intervention |
| Tao et al, 2018^10^ | Ineligible intervention |
| Mo et al, 2021^11^ | Ineligible intervention |
| Shao et al, 2016^12^ | Ineligible intervention |
| Wei et al, 2016^13^ | Ineligible intervention |
| Su 2019^14^ | Ineligible intervention |
| Su et al, 2018^15^ | Ineligible intervention |
| Wei et al, 2019^16^ | Ineligible intervention |
| Lu 2020^17^ | Ineligible control group |

References:

1. Liang RJ, Liang RK.Effects of integrated Traditional Chinese and Western medicine on cognitive function and mental behavior in Alzheimer's disease. J North Sichuan Med Colleg 2021,36(04):454-458.
2. Li XY, Gao M, Wang XZ, et al. Effect of Acupuncture Combined with Swallowing Electrical Stimulation on the Range of Motion of Hyolaryngeal Complex in Senile Dementia Coupled with Deglutition Disorder. Shanghai J Acu-mox 2020,39(10):1253-1258.
3. Yang S, Shen ZR. Clinical application of acupuncture and moxibustion combined with traditional Chinese medicine dialectical treatment of senile dementia.Medical diet therapy and health 2019(15):34-35.
4. Tony Willcox. The Effects of Acupuncture on Mild Cognitive Impairment(MCI) due to Alzheimer’s Disease(AD)[Doctor]. Anhui University of Chinese Medicine, 2016.
5. Chen W, Jiang LF, Wu Lin, et al. Clinical effect observation of integrated treatment of Chinese and western medicine for Alzheimer's disease. Intern Med Chin 2015,10(02):176-178+232.
6. Chen Q, Gu Q. Observation on the curative effect of combining the method of filling the essence and promoting blood circulation with acupoint application in the treatment of Alzheimer's disease with essence deficiency and blood stasis. Contemporary Medicine Forum 2014, 12(20): 20-21.
7. Yuan FG, GuoZF.40 cases of senile dementia treated by abdominal acupuncture combined with electroacupuncture point .Guang Ming Zhong Yi 2013,28(06):1206-1207.
8. Ying J. Clinical Study on the Influence of Acupuncture on the Psychobehavioral Symptoms of Alzheimer's Disease [Master]. Chengdu University of Traditional Chinese Medicine, 2006.
9. Du M, Li HJ. To observe the effect of acupuncture and moxibustion on senile dementia and its effect on the metabolism of cytokines and blood lipid. China Health Care & Nutrition 2019;29(10) :1004-7484.
10. Tao SX, Li RH. Clinical efficacy and clinical value of acupuncture Baihui and Yongquan points in senile dementia treatment. J Front Med 2018;8(31): 2095-1752
11. Mo J, Huang LF, Fu XL. Effect of tianzhi granules combined with massage acupuncture on sleep quality of patients with Alzheimer's disease. Zhejiang J TCM 2021;56(02):101-02.
12. Shao ZJ. Analysis of the Value of Acupuncture Baihui, Yongquan acupoint in the treatment of senile dementia. China Continue Med Edu 2016;8(27):166-67.
13. Wei YX, Zhang BC, Zhou J, et al. Clinical effect of acupuncture and moxibustion Baihui and Yongquan points on senile dementia. Chin J Gerontol 2016;36(12):2916-17.
14. Su HF. Treatment of Senile Dementia by Acupuncture Plus Moxbustion Clinical Observation of Spleen and Kidney Deficiency Type. Heilongjiang University of Chinese Medicine, 2019.
15. Su QD, He XH. Therapeutic Efficacy Observation of Mild Moxibustion in Treating Alzheimer’s Disease. Shanghai J Acu-mox, 2018;37(06): 623-25.
16. Wei LF, Huang HH, Wu XQ, et al. Clinical Observation on the Effect of Thunder Fire Moxibustion on Early Senile Dementia. Pop Sci Tech, 2019, 21(03): 46-48.
17. Lu YQ. Clinical Study of Acupuncture Combined with Ear Acupuncture ON Sleep Disorders in Patients With Alzheimer's Disease Based on Dumai Theory [Master]. Heilongjiang University of Chinese Medicine, 2020.

**Table S3.**

(a) Node-splitting test result of improvement of MMSE.

| Name | Direct Effect | Indirect Effect | Overall | *P-Value* |
| --- | --- | --- | --- | --- |
| SA, SA+CM | 1.75 (-4.15, 7.25) | -0.34 (-4.24, 3.40) | 0.96 (-2.50, 4.27) | 0.52 |

Note: SA: scalp acupuncture; CM: conventional medicine; MMSE: the Mini-Mental State Examination.

(b) Node-splitting test result of improvement of ADL.

| Name | Direct Effect | Indirect Effect | Overall | *P-Value* |
| --- | --- | --- | --- | --- |
| SA, SA+CM | 5.90 (1.53, 9.74) | 0.29 (-3.19, 3.80) | 3.17 (-0.33, 6.73) | 0.05 |

Note: SA: scalp acupuncture; CM: conventional medicine; ADL: Activities of Daily Living.

**Table S4.**

(a) The PSRF value of improvement of MMSE.

| Parameter | PSRF |
| --- | --- |
| d.CM.EA | 1 |
| d.CM.EA+CM | 1 |
| d.CM.FA+CM | 1 |
| d.CM.MA | 1 |
| d.CM.MA+CM | 1 |
| d.CM.SA | 1 |
| d.CM.SA+CM | 1 |
| d.CM.WA+CM | 1 |
| d.MA.WA | 1 |
| sd.d | 1 |

(b) The PSRF value of reduction of ADAS-Cog.

| Parameter | PSRF |
| --- | --- |
| d.CM.EA+CM | 1 |
| d.CM.MA | 1 |
| d.CM.MA+CM | 1 |
| d.CM.SA | 1 |
| d.CM.SA+CM | 1 |
| sd.d | 1 |

Note: MA: manual acupuncture; EA: electro-acupuncture; SA: scalp acupuncture; CM: conventional medicine; ADAS-cog: the Alzheimer's Disease Assessment Scale-Cognitive.

(c) The PSRF value of response rate.

| Parameter | PSRF |
| --- | --- |
| d.CM.EA | 1.03 |
| d.CM.EA+CM | 1.03 |
| d.CM.FA+CM | 1.00 |
| d.CM.MA | 1.02 |
| d.CM.MA+CM | 1.01 |
| d.CM.SA | 1.03 |
| d.CM.SA+CM | 1.08 |
| d.CM.WA+CM | 1.07 |
| d.MA.WA | 1.05 |
| sd.d | 1.03 |

Note: MA: manual acupuncture; EA: electro-acupuncture; FA: fire acupuncture; WA: warm acupuncture; SA: scalp acupuncture; CM: conventional medicine.

(d) The PSRF value of improvement of ADL.

| Parameter | PSRF |
| --- | --- |
| d.CM.EA | 1 |
| d.CM.EA+CM | 1 |
| d.CM.FA+CM | 1 |
| d.CM.MA | 1 |
| d.CM.MA+CM | 1 |
| d.CM.SA | 1 |
| d.CM.SA+CM | 1 |
| d.MA.WA | 1 |
| sd.d | 1 |

**Table S5. Quality of evidence of improvement of MMSE in network meta-analysis.**

| Comparison | | | Quality of evidence | | |
| --- | --- | --- | --- | --- | --- |
|  |  |  | Direct evidence | Indirect evidence | Network meta-analysis |
| EA+CM | VS | FA+CM | - | Critically Low | Critically Low |
| EA+CM | VS | SA+CM | - | Critically Low | Critically Low |
| EA+CM | VS | SA | - | Critically Low | Critically Low |
| EA+CM | VS | EA | - | Critically Low | Critically Low |
| EA+CM | VS | MA+CM | - | Critically Low | Critically Low |
| EA+CM | VS | MA | - | Critically Low | Critically Low |
| EA+CM | VS | WA | - | Critically Low | Critically Low |
| EA+CM | VS | WA+CM | - | Critically Low | Critically Low |
| EA+CM | VS | CM | Critically Low | Critically Low | Critically Low |
| FA+CM | VS | SA+CM | - | Critically Low | Critically Low |
| FA+CM | VS | SA | - | Critically Low | Critically Low |
| FA+CM | VS | EA | - | Critically Low | Critically Low |
| FA+CM | VS | MA+CM | - | Critically Low | Critically Low |
| FA+CM | VS | MA | - | Critically Low | Critically Low |
| FA+CM | VS | WA | - | Critically Low | Critically Low |
| FA+CM | VS | WA+CM | - | Critically Low | Critically Low |
| FA+CM | VS | CM | Critically Low | Critically Low | Critically Low |
| SA+CM | VS | SA | Critically Low | Critically Low | Critically Low |
| SA+CM | VS | EA | - | Critically Low | Critically Low |
| SA+CM | VS | MA+CM | - | Critically Low | Critically Low |
| SA+CM | VS | MA | - | Critically Low | Critically Low |
| SA+CM | VS | WA | - | Critically Low | Critically Low |
| SA+CM | VS | WA+CM | - | Critically Low | Critically Low |
| SA+CM | VS | CM | Critically Low | Critically Low | Critically Low |
| SA | VS | EA | - | Critically Low | Critically Low |
| SA | VS | MA+CM | - | Critically Low | Critically Low |
| SA | VS | MA | - | Critically Low | Critically Low |
| SA | VS | WA | - | Critically Low | Critically Low |
| SA | VS | WA+CM | - | Critically Low | Critically Low |
| SA | VS | CM | Critically Low | Critically Low | Critically Low |
| EA | VS | MA+CM | - | Critically Low | Critically Low |
| EA | VS | MA | - | Critically Low | Critically Low |
| EA | VS | WA | - | Critically Low | Critically Low |
| EA | VS | WA+CM | - | Critically Low | Critically Low |
| EA | VS | CM | Critically Low | Critically Low | Critically Low |
| MA+CM | VS | MA | - | Critically Low | Critically Low |
| MA+CM | VS | WA | - | Critically Low | Critically Low |
| MA+CM | VS | WA+CM | - | Critically Low | Critically Low |
| MA+CM | VS | CM | Critically Low | Critically Low | Critically Low |
| MA | VS | WA | Critically Low | Critically Low | Critically Low |
| MA | VS | WA+CM | - | Critically Low | Critically Low |
| MA | VS | CM | Critically Low | Critically Low | Critically Low |
| WA | VS | WA+CM | - | Critically Low | Critically Low |
| WA | VS | CM | - | Critically Low | Critically Low |
| WA+CM | VS | CM | Critically Low | Critically Low | Critically Low |

**Table S6.** Quality of evidence of reduction of ADAS-Cog in network meta-analysis.

| Comparison | | | Quality of evidence | | |
| --- | --- | --- | --- | --- | --- |
|  |  |  | Direct evidence | Indirect evidence | Network meta-analysis |
| SA+CM | VS | EA+CM | - | Critically Low | Critically Low |
| SA+CM | VS | MA+CM | - | Critically Low | Critically Low |
| SA+CM | VS | SA | Critically Low | Critically Low | Critically Low |
| SA+CM | VS | MA | - | Critically Low | Critically Low |
| SA+CM | VS | CM | Critically Low | Critically Low | Critically Low |
| EA+CM | VS | MA+CM | - | Critically Low | Critically Low |
| EA+CM | VS | SA | - | Critically Low | Critically Low |
| EA+CM | VS | MA | - | Critically Low | Critically Low |
| EA+CM | VS | CM | Critically Low | Critically Low | Critically Low |
| MA+CM | VS | SA | - | Critically Low | Critically Low |
| MA+CM | VS | MA | - | Critically Low | Critically Low |
| MA+CM | VS | CM | Low | Critically Low | Low |
| SA | VS | MA | - | Critically Low | Critically Low |
| SA | VS | CM | Critically Low | Critically Low | Critically Low |
| MA | VS | CM | Low | Critically Low | Low |

**Table S7.** Quality of evidence of response rate in network meta-analysis.

| Comparison | | | Quality of evidence | | |
| --- | --- | --- | --- | --- | --- |
|  |  |  | Direct evidence | Indirect evidence | Network meta-analysis |
| FA+CM | VS | SA+CM | - | Critically Low | Critically Low |
| FA+CM | VS | SA | - | Critically Low | Critically Low |
| FA+CM | VS | EA | - | Critically Low | Critically Low |
| FA+CM | VS | MA+CM | - | Critically Low | Critically Low |
| FA+CM | VS | MA | - | Critically Low | Critically Low |
| FA+CM | VS | WA | - | Critically Low | Critically Low |
| FA+CM | VS | WA+CM | - | Critically Low | Critically Low |
| FA+CM | VS | EA+CM | - | Critically Low | Critically Low |
| FA+CM | VS | CM | Critically Low | Critically Low | Critically Low |
| SA+CM | VS | SA | Critically Low | Critically Low | Critically Low |
| SA+CM | VS | EA | - | Critically Low | Critically Low |
| SA+CM | VS | MA+CM | - | Critically Low | Critically Low |
| SA+CM | VS | MA | - | Critically Low | Critically Low |
| SA+CM | VS | WA | - | Critically Low | Critically Low |
| SA+CM | VS | WA+CM | - | Critically Low | Critically Low |
| SA+CM | VS | EA+CM | - | Critically Low | Critically Low |
| SA+CM | VS | CM | Critically Low | Critically Low | Critically Low |
| SA | VS | EA | - | Critically Low | Critically Low |
| SA | VS | MA+CM | - | Critically Low | Critically Low |
| SA | VS | MA | - | Critically Low | Critically Low |
| SA | VS | WA | - | Critically Low | Critically Low |
| SA | VS | WA+CM | - | Critically Low | Critically Low |
| SA | VS | EA+CM | - | Critically Low | Critically Low |
| SA | VS | CM | Critically Low | Critically Low | Critically Low |
| EA | VS | MA+CM | - | Critically Low | Critically Low |
| EA | VS | MA | - | Critically Low | Critically Low |
| EA | VS | WA | - | Critically Low | Critically Low |
| EA | VS | WA+CM | - | Critically Low | Critically Low |
| EA | VS | EA+CM | - | Critically Low | Critically Low |
| EA | VS | CM | Critically Low | Critically Low | Critically Low |
| MA+CM | VS | MA | - | Critically Low | Critically Low |
| MA+CM | VS | WA | - | Critically Low | Critically Low |
| MA+CM | VS | WA+CM | - | Critically Low | Critically Low |
| MA+CM | VS | EA+CM | - | Critically Low | Critically Low |
| MA+CM | VS | CM | Critically Low | Critically Low | Critically Low |
| MA | VS | WA | Critically Low | Critically Low | Critically Low |
| MA | VS | WA+CM | - | Critically Low | Critically Low |
| MA | VS | EA+CM | - | Critically Low | Critically Low |
| MA | VS | CM | Low | Critically Low | Critically Low |
| WA | VS | WA+CM | - | Critically Low | Critically Low |
| WA | VS | EA+CM | - | Critically Low | Critically Low |
| WA | VS | CM | - | Critically Low | Critically Low |
| WA+CM | VS | EA+CM | - | Critically Low | Critically Low |
| WA+CM | VS | CM | Critically Low | Critically Low | Critically Low |
| EA+CM | VS | CM | Low | Critically Low | Low |

Table S8. Quality of evidence of improvement of ADL in network meta-analysis.

| Comparison | | | Quality of evidence | | |
| --- | --- | --- | --- | --- | --- |
|  |  |  | Direct evidence | Indirect evidence | Network meta-analysis |
| EA+CM | VS | SA+CM | - | Critically Low | Critically Low |
| EA+CM | VS | MA+CM | - | Critically Low | Critically Low |
| EA+CM | VS | FA+CM | - | Critically Low | Critically Low |
| EA+CM | VS | MA | - | Critically Low | Critically Low |
| EA+CM | VS | SA | - | Critically Low | Critically Low |
| EA+CM | VS | EA | - | Critically Low | Critically Low |
| EA+CM | VS | WA | - | Critically Low | Critically Low |
| EA+CM | VS | CM | Critically Low | Critically Low | Critically Low |
| SA+CM | VS | MA+CM | - | Critically Low | Critically Low |
| SA+CM | VS | FA+CM | - | Critically Low | Critically Low |
| SA+CM | VS | MA | - | Critically Low | Critically Low |
| SA+CM | VS | SA | Critically Low | Critically Low | Critically Low |
| SA+CM | VS | EA | - | Critically Low | Critically Low |
| SA+CM | VS | WA | - | Critically Low | Critically Low |
| SA+CM | VS | CM | Critically Low | Critically Low | Critically Low |
| MA+CM | VS | FA+CM | - | Critically Low | Critically Low |
| MA+CM | VS | MA | - | Critically Low | Critically Low |
| MA+CM | VS | EA | - | Critically Low | Critically Low |
| MA+CM | VS | SA | - | Critically Low | Critically Low |
| MA+CM | VS | WA | - | Critically Low | Critically Low |
| MA+CM | VS | CM | Low | Critically Low | Low |
| FA+CM | VS | MA | - | Critically Low | Critically Low |
| FA+CM | VS | EA | - | Critically Low | Critically Low |
| FA+CM | VS | SA | - | Critically Low | Critically Low |
| FA+CM | VS | WA | - | Critically Low | Critically Low |
| FA+CM | VS | CM | Critically Low | Critically Low | Critically Low |
| MA | VS | SA | - | Critically Low | Critically Low |
| MA | VS | EA | - | Critically Low | Critically Low |
| MA | VS | WA | Critically Low | Critically Low | Critically Low |
| MA | VS | CM | Critically Low | Critically Low | Critically Low |
| SA | VS | EA | - | Critically Low | Critically Low |
| SA | VS | WA | - | Critically Low | Critically Low |
| SA | VS | CM | Critically Low | Critically Low | Critically Low |
| EA | VS | WA | - | Critically Low | Critically Low |
| EA | VS | CM | Critically Low | Critically Low | Critically Low |
| WA | VS | CM | Critically Low | Critically Low | Critically Low |
